# Elucidating Mechanisms of Tolerance to *Salmonella* Typhimurium across Long-Term Infections Using the Collaborative Cross

**DOI:** 10.1128/mbio.01120-22

**Published:** 2022-07-26

**Authors:** Kristin Scoggin, Jyotsana Gupta, Rachel Lynch, Aravindh Nagarajan, Manuchehr Aminian, Amy Peterson, L. Garry Adams, Michael Kirby, David W. Threadgill, Helene L. Andrews-Polymenis

**Affiliations:** a Interdisciplinary Program in Genetics, Texas A&M University, College Station, Texas, USA; b Department of Molecular and Cellular Medicine, Texas A&M University, College Station, Texas, USA; c Department of Microbial Pathogenesis and Immunology, Texas A&M University, College Station, Texas, USA; d Department of Mathematics, Colorado State University, Fort Collins, Colorado, USA, USA; e Department of Veterinary Pathobiology, College of Veterinary Medicine, Texas A&M, College Station, Texas, USA; f Texas A&M Institute for Genome Sciences and Society, Texas A&M University, College Station, Texas, USA; g Department of Biochemistry and Biophysics and Department of Nutrition, Texas A&M University, College Station Texas, USA; University of Michigan—Ann Arbor

**Keywords:** collaborative cross, fibrinogen, QTL, *Salmonella*, resistance, tolerance

## Abstract

Understanding the molecular mechanisms underlying resistance and tolerance to pathogen infection may present the opportunity to develop novel interventions. Resistance is the absence of clinical disease with a low pathogen burden, while tolerance is minimal clinical disease with a high pathogen burden. *Salmonella* is a worldwide health concern. We studied 18 strains of collaborative cross mice that survive acute *Salmonella* Typhimurium (STm) infections. We infected these strains orally and monitored them for 3 weeks. Five strains cleared STm (resistant), six strains maintained a bacterial load and survived (tolerant), while seven strains survived >7 days but succumbed to infection within the study period and were called “delayed susceptible.” Tolerant strains were colonized in the Peyer’s patches, mesenteric lymph node, spleen, and liver, while resistant strains had significantly reduced bacterial colonization. Tolerant strains had lower preinfection core body temperatures and had disrupted circadian patterns of body temperature postinfection sooner than other strains. Tolerant strains had higher circulating total white blood cells than resistant strains, driven by increased numbers of neutrophils. Tolerant strains had more severe tissue damage and higher circulating levels of monocyte chemoattractant protein 1 (MCP-1) and interferon gamma (IFN-γ), but lower levels of epithelial neutrophil-activating protein 78 (ENA-78) than resistant strains. Quantitative trait locus (QTL) analysis revealed one significant association and six suggestive associations. Gene expression analysis identified 22 genes that are differentially regulated in tolerant versus resistant animals that overlapped these QTLs. Fibrinogen genes (*Fga*, *Fgb*, and *Fgg*) were found across the QTL, RNA, and top canonical pathways, making them the best candidate genes for differentiating tolerance and resistance.

## INTRODUCTION

Salmonella enterica is a Gram-negative bacterium that cause a range of diseases, including gastroenteritis, sepsis, and typhoid fever in various mammalian and avian hosts (1, 2). A total of 93.8 million cases nontyphoidal Salmonella (NTS) occur worldwide every year in humans, resulting in 155,000 deaths (3). The severity of an NTS infection depends on several factors, including host age, health, and genetics, as well as Salmonella serotype (4–6). Salmonella enterica serotype Typhimurium (STm) causes gastroenteritis in humans and can cause serious bacteremia. In mice, this serotype causes a fatal systemic infection in susceptible mouse strains that models invasive infections in humans (1, 7).

Various strains of inbred mice respond to STm infection differently. C57BL/6J and BALB/cJ have mutations in solute carrier family 11 member 1 (*Slc11a1*) and are highly susceptible to fatal STm bacteremia (8–10). Other strains, including 129 Sv, CBA/J, and A/J, are wild type at the *Slc11a1* locus and do not develop severe systemic salmonellosis (8–11). Other genes, including neutrophil cytosolic factor 2 (*Ncf2*), Toll-like receptor 4 (*Tlr4*), interferon gamma (*Ifng*), and histocompatibility complex (H2) haplotypes, have also been linked to survival after STm infections in mice (1, 12–15).

Since previous STm infection studies have used primarily traditionally inbred mouse strains, the host genetic diversity influencing STm infection in the mouse has not been thoroughly explored. The collaborative cross (CC) mouse population is a panel of recombinant inbred strains that recapitulate the genetic diversity found in human populations (16–19). The CC founders capture 90% of the genetic diversity found in the mouse genome and represents a wider range of phenotypes, including immune phenotypes, than traditional inbred strains (20–22). For example, strains with high viral titers after experimental infection with influenza virus or Ebola virus and good health status have been identified in the CC (23–25).

How a host responds to infection can be categorized as resistant or susceptible based on the host’s ability to clear the infection (1, 26). A third response called tolerance has been described in plant systems and is now being applied to other systems, including *Drosophila* and mice (27–33). In tolerance, the host has minimal signs of infection in the face of a high pathogen burden (32, 34, 35), and this quality has been attributed to the ability to limit damage (31). Genetic variation in both resistance and tolerance has been demonstrated using murine infections with plasmodium (31, 36). Our recent work with CC mice suggests that while there are multiple genes known to influence acute survival after STm infection, there are additional genes to be discovered that influence disease outcome after STm infection (37). Studies using the CC mouse population suggest that the disease outcomes after STm infection in mice are more complex than a binary susceptible and resistant classification (37).

To differentiate tolerance from resistance to Salmonella infections, 18 strains of CC mice that survived acute infection were orally infected with STm and monitored for up to 3 weeks (37). Our experiments revealed that some CC strains are tolerant to STm infection and that these strains maintain a significantly higher bacterial burden than resistant strains in Peyer’s patches (PP), mesenteric lymph nodes (MLN), spleen, and liver. Tolerant strains lost more weight than resistant strains despite surviving infection. Not surprisingly, resistant strains had significantly reduced tissue damage in the spleen and liver relative to tolerant strains. Tolerant strains had more circulating total white blood cells (WBCs), driven by higher circulating neutrophils, and higher serum interferon gamma (IFN-γ) and monocyte chemoattractant protein-1 (MCP-1) than resistant strains. Resistant strains had significantly more circulating epithelial neutrophil-activating protein 78 (ENA-78) than tolerant strains. Using quantitative trait locus (QTL) analysis, we identified one significant association, *Scq1*, and six suggestive associations. Transcriptome sequencing (RNA-seq) analysis allowed us to narrow these regions to 22 candidate genes, and the most highly differentially expressed were the fibrinogen subunits *Fga*, *Fgb*, and *Fgg*. RNA-seq analysis also allowed us to identify the top five canonical pathways, which also contained these fibrinogen genes.

## RESULTS

### Three-week *Salmonella* infections distinguish tolerant and resistant strains and reveal another phenotype: delayed susceptibility.

**FIG 1:**
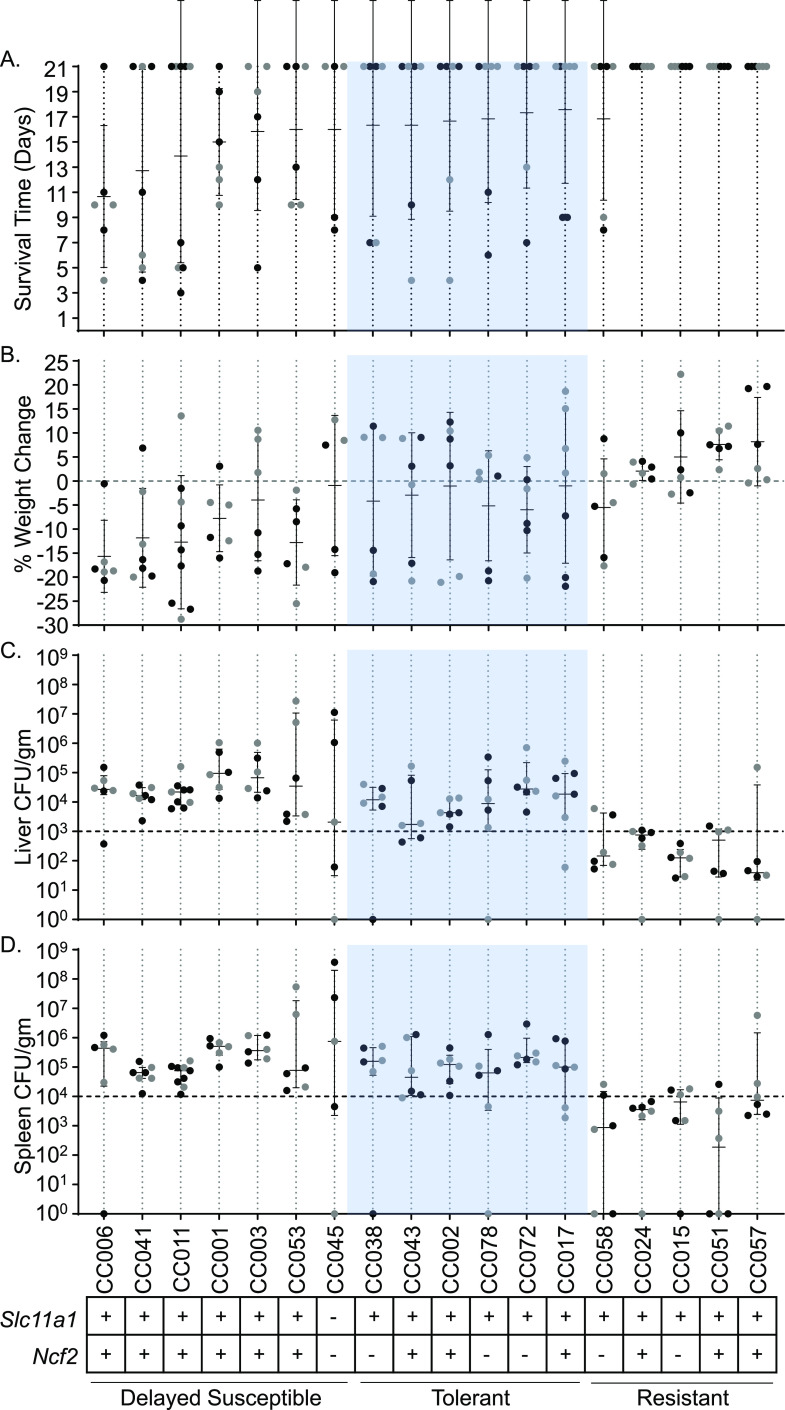
Delayed susceptible, tolerant, and resistant strains have distinct responses to STm infections. (A and B) Survival time (A) and percent weight change (B) after infection with STm. Strains are shown in ascending order of survival and weight change if survival was equal between strains. Means and standard deviations are shown. (C and D) Liver (C) and spleen (D) CFU counts per gram of organ at time of necropsy. Medians and interquartile ranges are shown. Circles represent individual mice (black circles represent males, and gray circles represent females). A total of 18 CC strains are represented. *Slc11a1* and *Ncf2* status (+, wild type; –, mutated) are shown at the bottom, as well as the response to infection (tolerant shown in blue).

Based on survival of acute STm infections (7 days) (37), 18 CC mouse strains were chosen to undergo a 3-week infection with STm ATCC 14028 to identify tolerant and resistant phenotypes. Tolerance was defined as strains that had at least 4/6 mice survive to day 7 during 1-week infections (37), at least 4/6 mice survive to day 21 during 3-week infections and had median colonizations of at least 10^3^ CFU/g in the liver and 10^4^ CFU/g in the spleen (Fig. 1). Resistant strains had the same or better survival as tolerant mice but had reduced bacterial burden with medians of <10^3^ CFU/g in the liver and <10^4^ CFU/g in the spleen (Fig. 1).

Of the 18 strains analyzed (see Table S4 in the supplemental material for complete strain names), 6 were categorized as tolerant (CC002, CC017, CC038, CC043, CC072, and CC078) and 5 were categorized as resistant (CC015, CC024, CC051, CC057, and CC058). The remaining seven strains did not fit into either the tolerant or resistant category because 3/6 or fewer mice survived the 3-week study period, despite having at least 4/6 mice survive our earlier acute study (37). We categorized these strains as “delayed susceptible” since they survive longer than susceptible strains.

10.1128/mbio.01120-22.10 TABLE S4 Numbers of mice used and facility origins for 18 CC strains. Download Table S4, PDF file, 0.01 MB. Copyright © 2022 Scoggin et al. 2022 Scoggin et al. https://creativecommons.org/licenses/by/4.0/ This content is distributed under the terms of the Creative Commons Attribution 4.0 International license.

Tolerant strains had mean survival times of 16 to 18 days (Fig. 1A). Four of the five resistant strains had all six mice survive to day 21. For the remaining resistant strain, CC058, 4/6 mice survived to day 21 with a mean survival of 16.83 days (Fig. 1A). Delayed susceptible strains had a mean survival time of 7 to 16 days (Fig. 1A). In general, both delayed susceptible and tolerant strains lost weight, while resistant strains gained weight. Delayed susceptible strains lost 0.93 to 15.67% of their starting weights while tolerant strains lost 1.02 to 5.97% (Fig. 1B). Resistant strains gained 2.05 to 8.17% of their starting weight, with the exception of one strain (CC058 lost 5.51%) (Fig. 1B).

We defined tolerance and resistance by a strain’s ability to control the bacterial burden in the spleen and liver. Resistant strains ranged in median colonization from 3.9 × 10^1^ to 7.53 × 10^2^ CFU/g in the liver and 1.88 × 10^2^ to 7.48 × 10^3^ CFU/g in the spleen (Fig. 1C and D; see also Fig. S6) and were statistically significantly more poorly colonized than tolerant strains. Tolerant strains ranged in median colonization from 1.74 × 10^3^ to 2.78 × 10^4^ CFU/g in the liver and 4.5 × 10^4^ to 2.13 × 10^5^ CFU/g in the spleen (Fig. 1C and D). Delayed susceptible strains overlapped with the tolerant median colonization ranges for both organs, 2.07 × 10^3^ to 9.44 × 10^4^ CFU/g in the liver and 6.5 × 10^4^ to 7.5 × 10^5^ CFU/g in the spleen (Fig. 1C and D). Overlapping ranges of colonization of tolerant and delayed susceptible strains demonstrate that tolerant strains survive a bacterial burden that is fatal for other CC strains. One tolerant strain, CC072, had the highest colonization of all tolerant strains in both the spleen and the liver, while another, CC043, was more poorly colonized in both organs, illustrating that there is a broad spectrum of bacterial colonization that is tolerated across host genetics. Thus, based on bacterial colonization and survival, we are able to define three categories of phenotypes: delayed sensitivity, tolerance, and resistance to STm infection in mice of diverse genetics.

10.1128/mbio.01120-22.6 FIG S6 Liver and spleen colonization is significantly lower in resistant strains at 3 weeks postinfection. (A and B) Liver (A) and spleen (B) CFU per gram of organ at the time of necropsy. Each circle represents an individual mouse, and medians and interquartile ranges are indicated. T, tolerant (open circles); R, resistant (closed circles); 1W, 1 week postinfection; 3W, 3 weeks postinfection. Eleven strains are included in the graph. A Kruskal-Wallis test was performed to identify significant differences (*, *P* < 0.05; **, *P* < 0.01; ***, *P* < 0.001; ****, *P* < 0.0001). Download FIG S6, TIF file, 2.5 MB. Copyright © 2022 Scoggin et al. 2022 Scoggin et al. https://creativecommons.org/licenses/by/4.0/ This content is distributed under the terms of the Creative Commons Attribution 4.0 International license.

### Tolerant strains maintain a higher bacterial load in Peyer’s patches and mesenteric lymph nodes than resistant strains.

**FIG 2:**
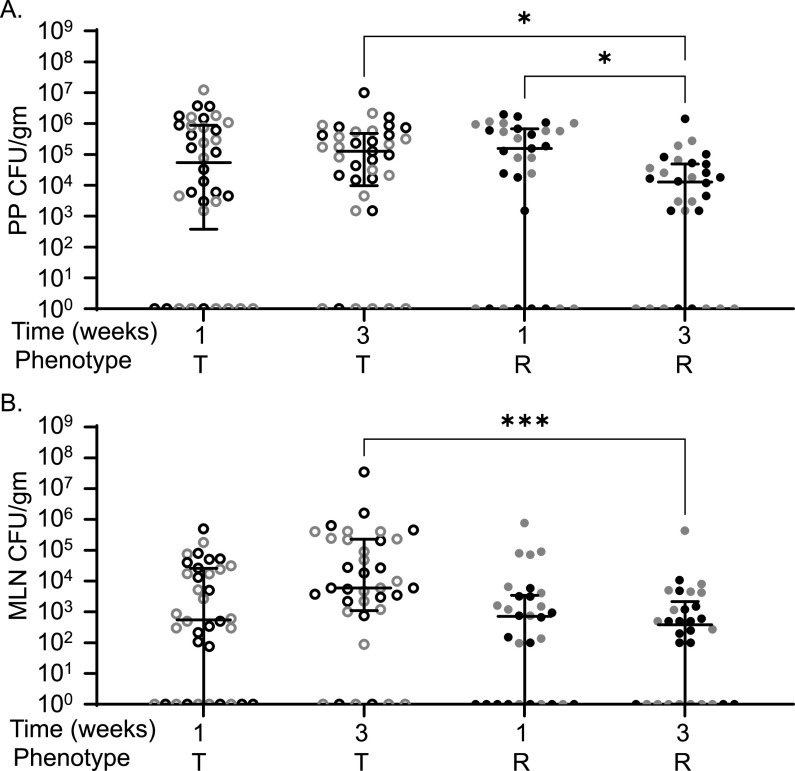
CC strains resistant to STm infection have lower MLN and PP colonization compared to tolerant strains. Peyer’s patches (PP) (A) and MLN (B) CFU counts per gram of organ at necropsy. Each circle represents an individual mouse (black circles represent males, and gray circles represent females), and medians and interquartile range, are indicated by lines. Tolerant (T, open circles) and resistant (R, filled circles) are indicated. A Kruskal-Wallis test was performed to identify significant differences (*, *P* < 0.05; **, *P* < 0.01; ***, *P* < 0.001).

While our initial focus was on spleen and liver, the ileum, cecum, colon, MLN, and PP were also examined for bacterial load after STm infection. Bacterial burden in the ileum, cecum, and colon was not significantly different in tolerant or resistant strains at 1 or 3 weeks postinfection (see Fig. S1, *P = *0.5600, *P *> 0.9999, and *P *> 0.9999). Tolerant and resistant strains were colonized similarly in their PP and MLN during acute (1 week) infections in previous experiments (Fig. 2; *P* > 0.9999) (37). At 3 weeks postinfection, tolerant strains remained more highly colonized than resistant strains in both PP (1.28 × 10^5^ versus 1.28 × 10^4^ CFU/g, *P = *0.038) and MLN (6.00 × 10^3^ versus 3.87 × 10^2^ CFU/g, *P = *0.0008) (Fig. 2). Tolerant strains were stably colonized in PP and MLN between 1 and 3 weeks postinfection, suggesting that they were unable to clear STm from these niches (Fig. 2). Resistant strains, however, reduced PP colonization significantly between 1 and 3 weeks postinfection (Fig. 2A, 1.56 × 10^5^ CFU/g and 1.28 × 10^4^ CFU/g, *P = *0.0451), while MLN remained stably colonized. Thus, during longer-term infections, resistant strains reduce STm colonization of the PP, while tolerant strains do not, and resistant strains maintain low levels of MLN colonization.

10.1128/mbio.01120-22.1 FIG S1 Ileum (A), cecum (B), and colon (C) CFU/g levels are not significantly different between tolerant and resistant strains. The CFU per gram values for each organ were determined at necropsy. Each circle represents an individual mouse, and medians and interquartile ranges are indicated. T, tolerant (open circles); R, resistant (closed circles). Eleven strains are included in the graph. Download FIG S1, TIF file, 2.5 MB. Copyright © 2022 Scoggin et al. 2022 Scoggin et al. https://creativecommons.org/licenses/by/4.0/ This content is distributed under the terms of the Creative Commons Attribution 4.0 International license.

### Tolerant strains have lower baseline core body temperature and deviate from their normal circadian pattern quickly postinfection.

**FIG 3:**
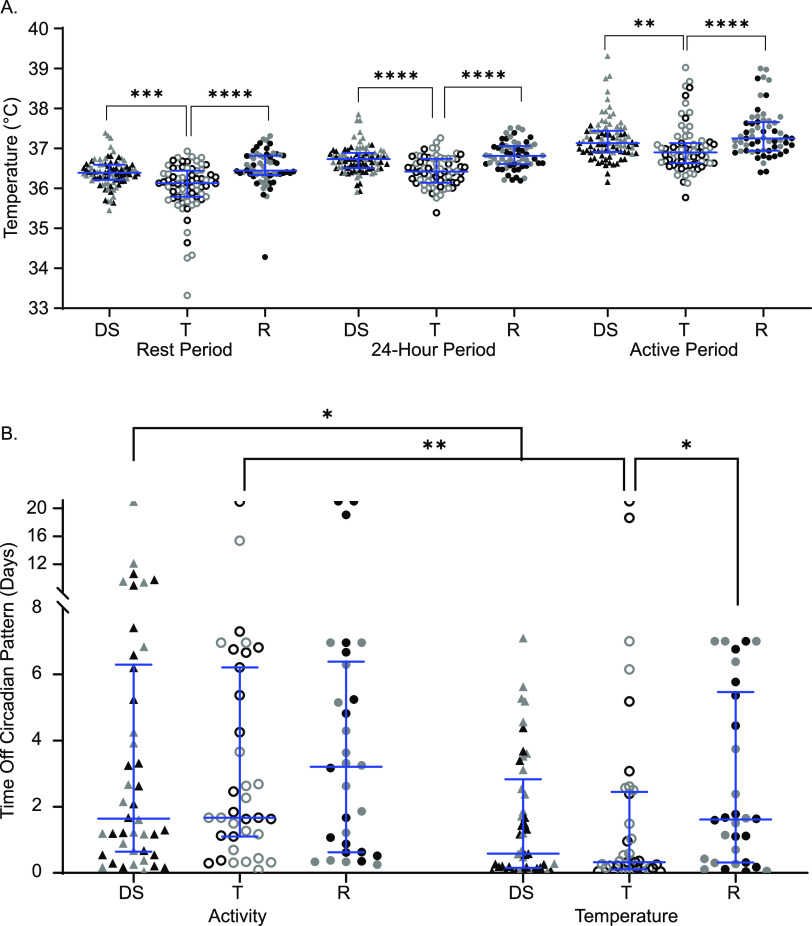
Tolerant mice have cooler body temperatures and deviate from normal body temperature patterns sooner than resistant mice. (A) Core body temperatures during resting, 24-h, and active periods for delayed susceptible (DS, filled triangles), tolerant (T, open circles), and resistant (R, filled circles) mice. (B) Times to deviation from circadian pattern (“off pattern”) for temperature and activity. Each symbol represents an individual mouse (black symbols represent males, and gray symbols represent females). Medians and interquartile ranges are indicated by blue lines. A Kruskal-Wallis test was performed to identify significant differences (*, *P* < 0.05; **, *P* < 0.01; ***, *P* < 0.001; ****, *P* < 0.0001).

Mice were implanted with telemetry devices that tracked temperature and activity continuously. Baseline measurements were taken for 1 week preinfection and for up to 3 weeks postinfection. The body temperature minimum corresponds to the minimum core body temperature reached during the “rest period” of the mouse (light hours), the maximum corresponds to the maximum core body temperature reached during the “active period” (dark hours), and the median describes the median core body temperature in a 24-h period. Tolerant strains had significantly lower baseline body temperatures when uninfected than both resistant and delayed susceptible strains across all three measurement periods (Fig. 3A; see also Table S1 in the supplemental material [rest, active, and 24-h periods]).

10.1128/mbio.01120-22.7 TABLE S1 Median values of core body temperature for rest period, 24-h period, and active period. Download Table S1, TIF file, 2.5 MB. Copyright © 2022 Scoggin et al. 2022 Scoggin et al. https://creativecommons.org/licenses/by/4.0/ This content is distributed under the terms of the Creative Commons Attribution 4.0 International license.

Since body temperature can be altered by differences in activity, activity levels were also analyzed for the fraction of time the mouse was active throughout a given time period. The fraction of time the mouse was active for their rest period, active period, and an entire 24-h period was not significantly different between these groups (see Fig. S2).

10.1128/mbio.01120-22.2 FIG S2 Preinfection activity levels are not significantly different between groups. The fractions of time active (0 to 1) for resting, 24-h, and active periods for delayed susceptible (DS [filled triangles]), tolerant (T [open circles]), and resistant (R [filled circles]) mice are indicated. Medians and interquartile ranges are indicated. Download FIG S2, TIF file, 2.5 MB. Copyright © 2022 Scoggin et al. 2022 Scoggin et al. https://creativecommons.org/licenses/by/4.0/ This content is distributed under the terms of the Creative Commons Attribution 4.0 International license.

Gross alterations in circadian patterns of core body temperature and locomotor activity occurred in nearly all infected lines of CC mice upon infection, but with various times of onset and various severities. STm tolerant mice deviated from their normal pattern of core body temperature very quickly, with a median of 463 min (7.7 h postinfection) (Fig. 3B) and without showing other perceptible clinical signs of disease. Delayed susceptible strains also deviated from their normal pattern of body temperature rapidly (835 min, ~13 h). Resistant mice maintained their circadian pattern significantly longer than tolerant mice with a median of 2,324.5 min (1.61 days) (Fig. 3B; *P* = 0.0458), but despite their resistance to infection, they had perceptible changes in the regulation of their core body temperature.

All groups maintained their circadian pattern of locomotor activity for a similar amounts of time: between 1 and 3 days postinfection (Fig. 3B). When the times to deviation from activity and temperature patterns (time to “off-pattern”) were compared for each group, both delayed susceptible and tolerant mice had disruptions in their temperature patterns sooner than disruptions in their activity patterns, suggesting that one does not immediately influence the other (Fig. 3B). Delayed susceptible mice deviated from their temperature pattern a median of 1,523.5 min (1.06 days) earlier than their activity pattern (Fig. 3B; *P* = 0.0351), while tolerant mice deviated from their temperature patterns a median of 1,940 min (1.34 days) earlier (Fig. 3B; *P* = 0.0031). Disrupted circadian patterns of temperature and activity were not significantly different in resistant mice (Fig. 3B; *P* > 0.9999). For delayed susceptible and tolerant mice, changes in core body temperature pattern signaled earlier changes in health than did changes in activity level, a finding consistent with our previous reports (37). Furthermore, tolerant strains appear to have a lower core baseline body temperature prior to infection than CC strains of other phenotypes. Postinfection, tolerant lines deviated from their circadian pattern of body temperature the fastest.

### Tolerant strains have increased total white blood cell counts, driven by circulating neutrophils, relative to resistant strains.

**FIG 4:**
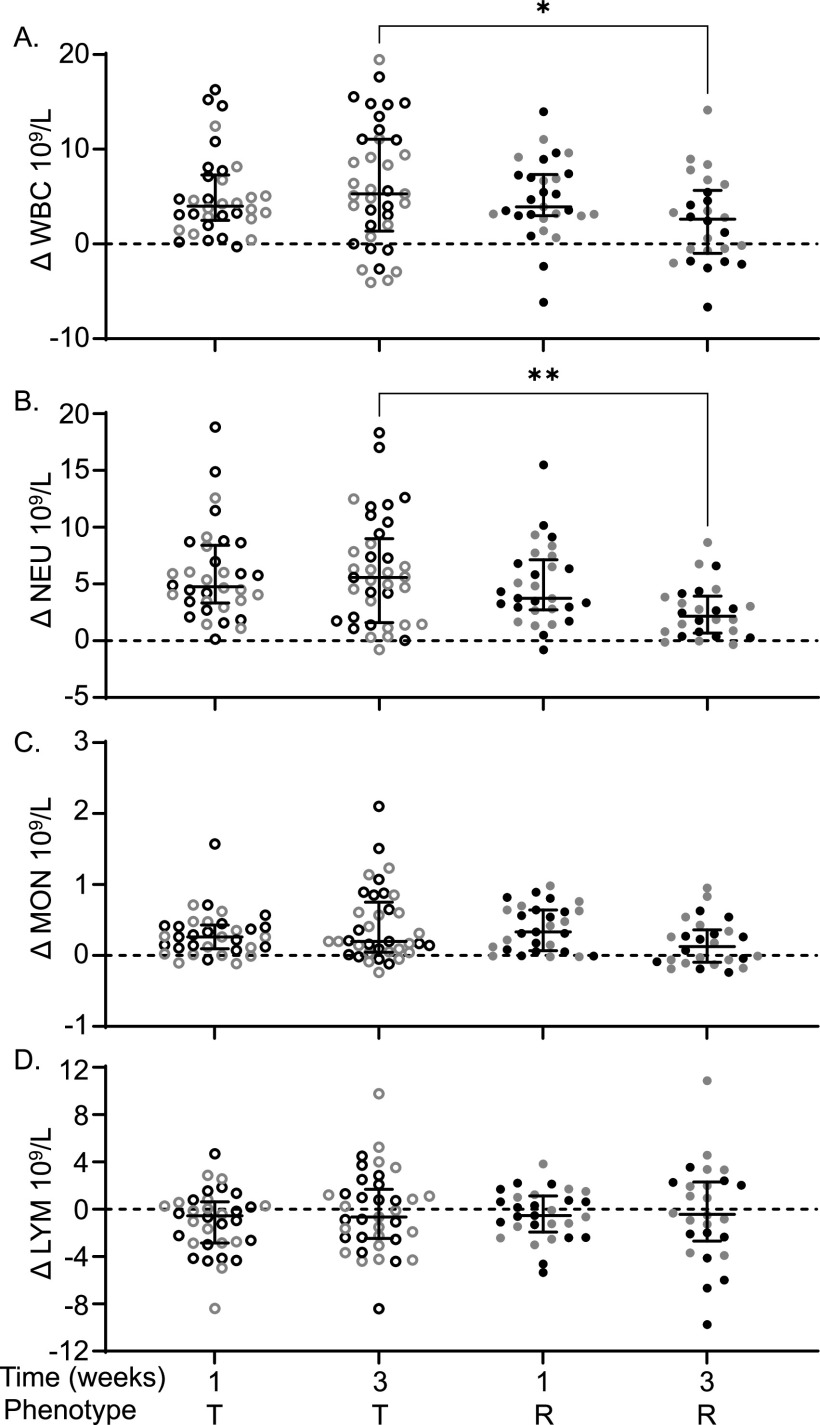
Tolerant mice have higher WBC and higher NEU than resistant mice. Difference (infected – uninfected = Δ) between infected and uninfected for circulating. (A) Total white blood cells (WBC); (B) neutrophils (NEU); (C) monocytes (MON); (D) lymphocytes (LYM). Each circle represents an individual mouse (black circles represent males, and gray circles represent females), and lines represent medians and interquartile ranges. T, tolerant (open circles); R, resistant (filled circles). A Kruskal-Wallis test was performed to identify significant differences (*, *P* < 0.05; **, *P* < 0.01).

Complete blood counts (CBC) were performed preinfection and at the time of necropsy for mice that were euthanized at 1 week or 3 weeks postinfection (37). The difference between preinfection and postinfection CBC components was calculated to determine the change (Δ) in each component (see Table S2A). We included only tolerant and resistant strains in this analysis because they were euthanized at the same time point. At 3 weeks postinfection, tolerant mice had significantly more circulating white blood cells (WBC) than resistant mice (5.29 × 10^9^/L versus 2.61 × 10^9^/L, Fig. 4A; *P* = 0.0396). The differential count suggested that circulating neutrophils (NEU) were elevated in tolerant mice relative to resistant mice at 3 weeks postinfection (Fig. 4B; 5.58 × 10^9^/L versus 2.16 × 10^9^/L, *P = *0.0029). Circulating monocyte (MON) and lymphocyte (LYM) numbers were similar between tolerant and resistant mice (Fig. 4C and D; *P* = 0.2278, > 0.9999). Thus, circulating neutrophils in tolerant mice remain elevated for the duration of the study period.

10.1128/mbio.01120-22.8 TABLE S2 (A) Complete blood counts from infected mice taken at necropsy minus uninfected means for respective 18 CC strains. Means and standard deviations are shown. (B) A total of 36 cytokines were obtained from infected mice taken at necropsy minus uninfected means for the respective 18 CC strains. Means and standard deviations are shown. Download Table S2, XLSX file, 0.03 MB. Copyright © 2022 Scoggin et al. 2022 Scoggin et al. https://creativecommons.org/licenses/by/4.0/ This content is distributed under the terms of the Creative Commons Attribution 4.0 International license.

### At 3 weeks postinfection, tolerant mice have increased MCP-1 and IFN-γ levels and reduced ENA-78 levels compared to resistant mice.

Chemokines, cytokines, and their receptors control the movement of cells of the immune system. The levels of 36 cytokines/chemokines in serum from uninfected and from 1 and 3 week postinfection CC mice were determined. The change (infected – uninfected = Δ) in circulating cytokine/chemokine levels between uninfected and infected animals was calculated (see Table S2B). We included only tolerant and resistant strains in this analysis because they were euthanized at the same time point, while delayed susceptible animals were euthanized earlier. STm tolerant and resistant mice had different levels of three cytokines at 3 weeks postinfection: MCP-1, IFN-γ, and ENA-78.

**FIG 5:**
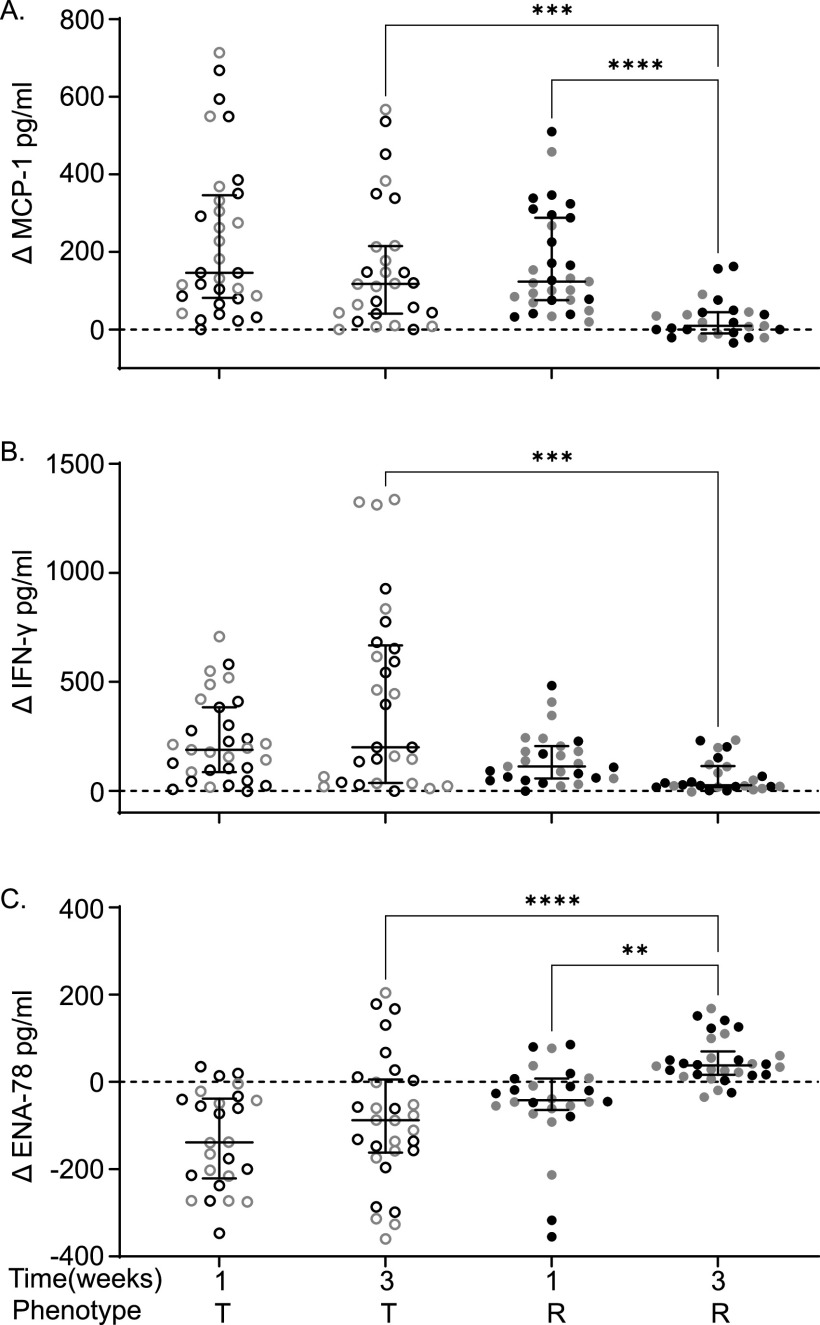
Tolerant strains have higher circulating MCP-1 and IFN-γ levels but a lower ENA-78 level at 3 weeks postinfection than resistant strains. Differences (Δ) between infected and uninfected levels of MCP-1 (A), IFN-γ (B), and ENA-78 (C) in tolerant and resistant strains. Circles represent individual mice (black circles represent males, and gray circles represent females), and lines represent medians and interquartile ranges. Outliers were removed. T, tolerant (open circles); R, resistant (closed circles). A Kruskal-Wallis test was performed to identify significant differences (*, *P* < 0.05; **, *P* < 0.01; ***, *P* < 0.001; ****, *P* < 0.0001).

MCP-1 (CCL2) is a key chemoattractant for monocytes and plays a critical role in their migration and tissue infiltration (38). The change in serum MCP-1 over baseline is similar for tolerant and resistant lines at 1 week postinfection. However, at 3 weeks postinfection, MCP-1 levels remain elevated in tolerant mice in response to infection, a median increase of 117.54 pg/mL, while circulating MCP-1 returns nearly to baseline in resistant mice (increase of 9.27 pg/mL over baseline [Fig. 5A; *P* = 0.0005]). Serum MCP-1 fell in resistant mice from a median increase of 123.57 pg/mL over baseline at 1 week postinfection to a median increase of 9.27 pg/mL at 3 weeks postinfection (Fig. 5A; *P* < 0.0001).

IFN-γ is vital for controlling intracellular replication of STm, promoting damage to the Salmonella containing vacuole, the intracellular niche of STm, and promoting production of reactive oxygen and nitrogen species from phagocytic cells (39). At 1 week postinfection, IFN-γ levels rose similarly in tolerant versus resistant mice. However, tolerant strains maintained a high level of circulating IFN-γ with a median increase of 200.04 pg/mL over baseline at 3 weeks postinfection, while resistant strains returned IFN-γ levels much closer to baseline (median increase of 25.99 pg/mL [Fig. 5B; *P* = 0.0001]).

ENA-78 (CXCL5) is a CXC motif chemokine normally produced by platelets that promotes accumulation of neutrophils (40). Tolerant strains had significantly lower circulating ENA-78 levels than STm resistant strains at 3 weeks postinfection compared to the baseline (Fig. 5C; −87.90 pg/mL versus 37.91 pg/mL, *P* = <0.0001). Resistant strains also significantly increased their ENA-78 levels from 1 to 3 weeks postinfection from −42.11 to 37.91 pg/mL over uninfected baseline (Fig. 5C; *P* = 0.0016). Thus, reduction of circulating CXCL5 appears to improve host defense to bacterial infection perhaps by allowing proper scavenging of other chemokines (keratinocyte-derived chemokine [KC or CXCL1] and macrophage inflammatory protein 2-alpha [MIP2-α or CXCL2]), allowing the formation of chemokine gradients and sensitizing CXCR2, resulting in an increased influx of neutrophils (41).

### Tissue damage is more severe in tolerant and delayed susceptible strains than in resistant strains.

Sections of the ileum, cecum, colon, spleen, and liver were stained with hematoxylin and eosin (H&E) and scored blindly by a board-certified pathologist using a previously described (37) scoring system of 0 to 4 (0 = normal, 4 = severe damage; see Table S3). Intestinal organs had minimal damage (scores 0 to 1) and were excluded from further analysis (see Fig. S3). The spleen and liver had a range of damage and were examined further at 1 and 3 weeks postinfection.

10.1128/mbio.01120-22.3 FIG S3 The ileum, cecum, and colon are minimally damaged. Ileum and cecum/colon histopathology means and standard deviations are shown for each group. Download FIG S3, TIF file, 2.5 MB. Copyright © 2022 Scoggin et al. 2022 Scoggin et al. https://creativecommons.org/licenses/by/4.0/ This content is distributed under the terms of the Creative Commons Attribution 4.0 International license.

10.1128/mbio.01120-22.9 TABLE S3 Histopathology scoring matrix with descriptions and pictures. The final score per organ corresponds to whichever section has the highest score. (A) Scoring matrix (from L. Garry Adams). (B) Representative images of spleen and liver histology scoring matrices. Tissues were sectioned and stained with H&E before being analyzed. Images are from L. Garry Adams. Download Table S3, PDF file, 0.2 MB. Copyright © 2022 Scoggin et al. 2022 Scoggin et al. https://creativecommons.org/licenses/by/4.0/ This content is distributed under the terms of the Creative Commons Attribution 4.0 International license.

**FIG 6:**
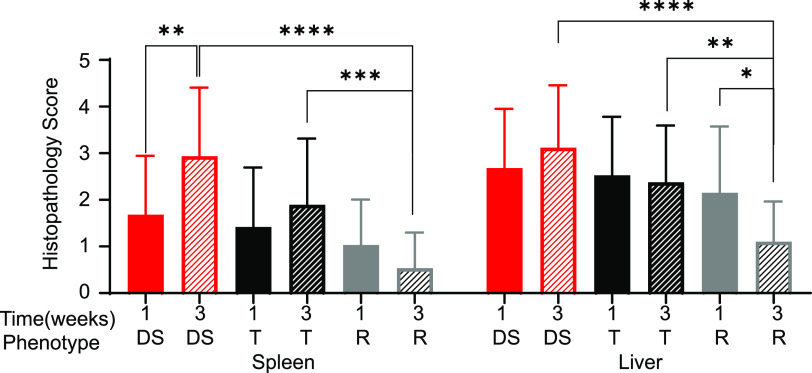
Resistant strains have less damage in the spleen and liver at 3 weeks postinfection than do tolerant and delayed susceptible strains. The mean spleen and liver histopathology scores for tissue damage and the standard deviations are indicated. DS, delayed susceptible (red bars), T, tolerant (black bars); R, resistant (gray bars). A Kruskal-Wallis test was performed to identify significant differences (*, *P* < 0.05; **, *P* < 0.01; ***, *P* < 0.001; ****, *P* < 0.0001).

When mice were grouped by response to infection, there was no significant difference between delayed susceptible, tolerant, and resistant for spleen or liver damage at 1 week postinfection. However, strains susceptible to acute infection had significantly more splenic damage at 7 days postinfection than strains of other phenotypes (see Fig. S4). At 3 weeks postinfection, however, significant differences became apparent. STm tolerant strains had a mean histopathology score of 1.89 ± 1.43; while delayed susceptible strains had a mean of 2.93 ± 1.47 for the spleen, damage to these tissues was not statistically significantly different (Fig. 6). Resistant strains had a mean of 0.53 ± 0.77 for the spleen, lower than for both tolerant and delayed susceptible strains (Fig. 6; *P* = 0.0004, <0.0001). Delayed susceptible strains also had increased spleen damage from 1.68 ± 1.26 at 1 week postinfection to 2.93 ± 1.47 at 3 weeks postinfection (Fig. 6; *P* = 0.007).

10.1128/mbio.01120-22.4 FIG S4 Susceptible strains have the most damage at 1 week postinfection compared to the other groups. The means spleen and liver histopathology scores for tissue damage and standard deviations are indicated. T, tolerant (black bars); R, resistant (gray bars); DS, delayed susceptible (red bars); S, susceptible (yellow bar); 1W, 1 week postinfection (filled bars). A Kruskal-Wallis test was performed to identify significant differences (*, *P* < 0.05; **, *P* < 0.01; ***, *P* < 0.001; ****, *P* < 0.0001). Download FIG S4, TIF file, 2.5 MB. Copyright © 2022 Scoggin et al. 2022 Scoggin et al. https://creativecommons.org/licenses/by/4.0/ This content is distributed under the terms of the Creative Commons Attribution 4.0 International license.

Although damage to the liver was more pronounced than splenic damage at 3 weeks postinfection, the trend was the same: tolerant strains suffered more severe damage than resistant strains (tolerant mean of 2.38 ± 1.22 and resistant mean of 1.10 ± 0.87; Fig. 6; *P* = 0.0058). Delayed susceptible strains also suffered more liver damage at 3 weeks postinfection than resistant strains (delayed susceptible mean of 3.12 ± 1.34 and resistant mean of 1.10 ± 0.87; Fig. 6, *P* < 0.0001). Liver damage was scored as resolving between 1 and 3 weeks postinfection in resistant strains (mean of 2.15 ± 1.42 at 1 week to 1.10 ± 0.87 at 3 weeks; Fig. 6, *P* = 0.0337).

To summarize, for those strains that survive acute infection, differences in the amount of tissue damage by phenotype are apparent by 3 weeks postinfection. Both tolerant and delayed susceptible strains displayed similar levels of tissue damage in spleen and liver, while the tissue damage in resistant strains was much reduced by the 3-week time point.

### Significant and suggestive genetic associations were identified across various phenotypes for 3 weeks post-STm infection.

**FIG 7:**
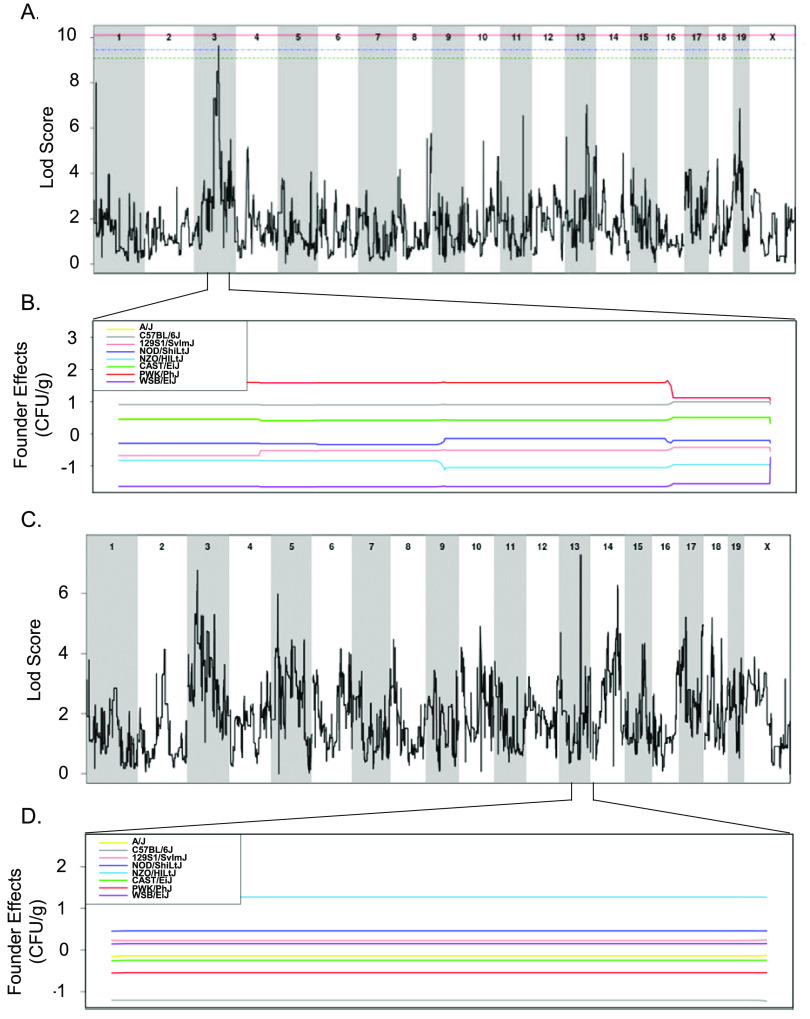
QTLs identified for spleen and liver colonization using 18 CC strains. (A and B) QTL of spleen CFU at 3 weeks postinfection (A) and allele effect plots for Chr 3 (B). (C and D) QTL of liver CFU at 3 weeks postinfection (C) and allele effect plots focused in on Chr 13 (D). The green dotted line indicates 85% significance, blue dotted line indicates 90% significance, and the red dotted line indicates 95% significance. Results were obtained using gQTL.

All 18 CC strains infected here were included in a quantitative trait locus (QTL) association of relevant phenotypes. A statistically significant association was identified for spleen colonization on mouse chromosome (Chr) 3 (spleen colonization QTL 1 [*Scq1*]) (Fig. 7 and Table 1). QTL *Scq1* contains 533 genes, with no obvious haplotype differences, likely due to the small number of strains in our analysis (Fig. 7A and B). A suggestive association was identified for liver colonization on Chr 13, containing 20 genes. The NZO founder has a high haplotype effect in this region, associated with an increase in liver colonization, and 3 CC strains carried this haplotype (CC001 [DS], CC003 [DS], and CC072 [T]) (Fig. 7C and D). Furthermore, the B6 founder strain has a low haplotype effect in this region, associated with reduced liver colonization, and 3 CC strains carried this haplotype (All resistant: CC015, CC057, and CC058) (Fig. 7C and D). Of the 20 genes in this region on Chr 13, none had single nucleotide polymorphism (SNP) differences matching the haplotype effects.

**TABLE 1:** QTL associations for spleen and liver colonization and resistance and tolerance categorization after STm infections^*a*^

QTL	Phenotype	Chr	LOD	*P*	Size (Mb)			Haplotype effect
					Proximal	Max	Distal	
	Resistance	3	8.44	2.07 × 10^−6^	95.34	95.94	100.14	High: NZO, 129S1, WSB
	Resistance	13	9.37	3.10 × 10^−7^	81.53	81.87	83.81	High: B6, PWK
	Resistance	17	6.85	4.88 × 10^−5^	86.91	88.97	90.81	High: PWK, NZO, CAST
	Tolerance	2	7.9	6.19 × 10^−6^	159.05	168.27	181.43	High NOD, NZO
	Tolerance	6	6.53	9.14 × 10^−5^	114.37	147.33	147.37	
*Scq1*	Spleen colonization	3	9.64	1.78 × 10^−7^	79.59	93.37	95.95	
	Liver colonization	13	7.28	2.11 × 10^−5^	81.83	83.66	83.8	High: NZO, low: B6

a A total of 18 CC strains were included in the spleen and liver colonization QTL, 32 CC strains were in the resistance QTL, and 27 CC strains were in the tolerance QTL. LOD, Logarithm of the odds; Chr, chromosome.

**FIG 8:**
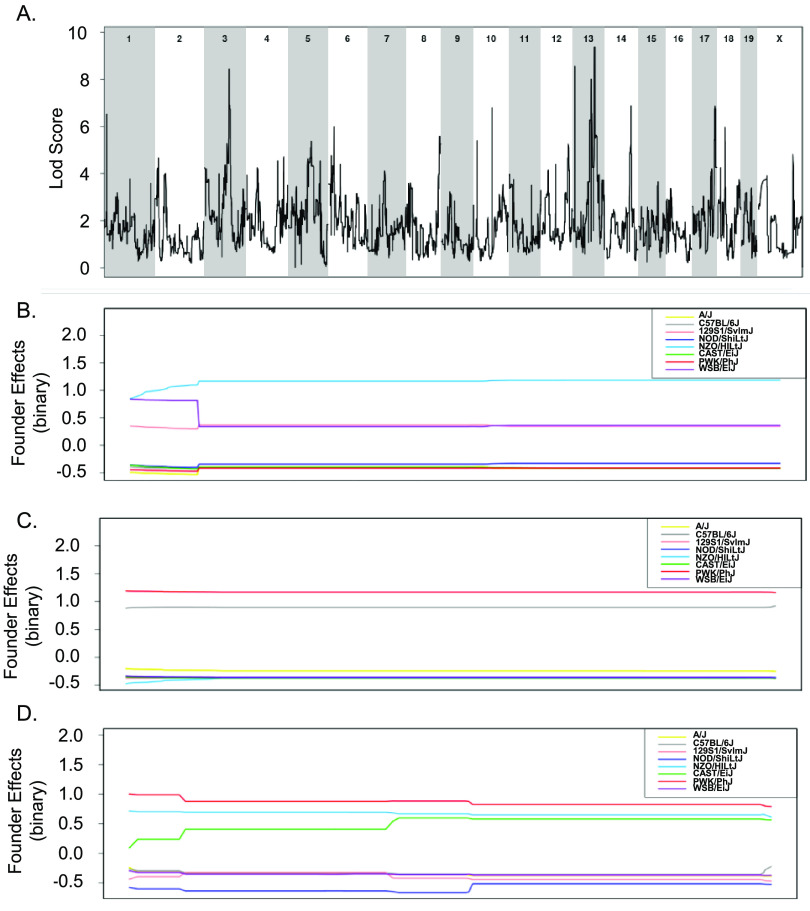
Binary categorization to identify QTLs linked to STm resistance using 32 CC strains. (A) QTL of resistant categorization (score of 1) versus susceptible, delayed susceptible, and tolerant categorization (score of 0) after STm infections. (B to D) Allele effect plots zoomed in for Chr 3 (B), Chr 13 (C), and Chr 17 (D). Results were obtained using gQTL.

To overcome the limitations of using small numbers of CC strains to explore the genes that distinguish tolerant and resistant strains, all 32 CC strains whose acute response to infection with STm is known were included in a binary QTL analysis (37). Resistant strains (CC015, CC024, CC051, CC057, and CC058) were assigned a value of 1 while the remaining tolerant, delayed susceptible, and susceptible strains were assigned a value of 0. This analysis identified three suggestive QTL on Chr 3, 13, and 17 (Fig. 8 and Table 1). The Chr 3 QTL contains 212 genes and high haplotype effects in NZO (All resistant: CC015 and CC058), 129S1 (CC024 [R], CC037 [S], CC053 [DS], and CC057 [R]), and WSB (CC051 [R]) suggested that regions from these founders conferred resistance (Fig. 8A and B). No SNP differences were identified in this region between NZO, 129S1, and WSB and the remaining CC founder strains.

The association on Chr 13 contained 29 genes and had a high haplotype effect (more STm resistant) for PWK (CC024 [R]) and B6 (CC015 [R], CC030 [S], CC057 [R], and CC058 [R]) (Fig. 8A and C). There were no genes in this region that had SNP differences that followed the haplotype effects. The association on Chr 17 contained 64 genes and had a high haplotype effect (more resistant to STm) for PWK (CC024 [R]), NZO (CC013 [S], CC051 [R], and CC057 [R]), and CAST (CC015 [R], CC019 [S], and CC058 [R]) (Fig. 8A and D). Only one gene had an SNP difference that corresponded to the haplotype effects.

**FIG 9:**
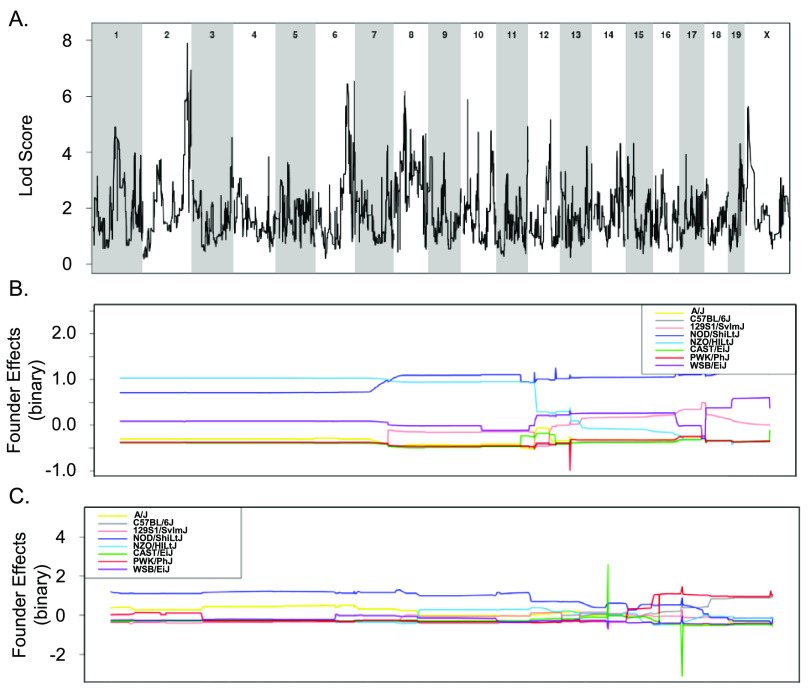
Binary categorization to identify QTLs linked to STm tolerance using 27 CC strains. (A) QTL of tolerant categorization (score of 1) versus susceptible and delayed susceptible categorization (score of 0) after STm infections. (B and C) Allele effect plots zoomed in for Chr 2 (B) and Chr 6 (C). Results were obtained using gQTL.

Tolerance to STm infection was also examined by assigning tolerant strains (CC002, CC017, CC038, CC043, CC072, and CC078) a score of 1 and susceptible and delayed susceptible strains a score of 0. A total of 27 CC strains were included in this analysis (resistant strains were excluded). This analysis yielded suggestive associations with QTL on Chr 2 and Chr 6 (Fig. 9 and Table 1). The associated region on Chr 2 contains 618 genes, and NOD (all tolerant: CC072 and CC078) and NZO (all tolerant: CC002 and CC043) had a high haplotype effect. Thus, strains that had a NOD or NZO allele at this location were more likely to be tolerant to STm infections (Fig. 9A and B). There were no genes in the Chr 2 region that had SNP differences corresponding to haplotype effects. The association on Chr 6 had 751 genes, but no haplotype effects were identified (Fig. 9A and C). While the experiments examining spleen and liver bacterial burdens did not contain enough strains to identify significant associations, the binary categorizations did not capture enough phenotypic diversity to be significant.

### Transcriptional differences in QTL regions in tolerant versus resistant mice.

RNA-seq analyses on spleen samples of uninfected and infected mice identified differentially expressed genes. Samples from infected animals were normalized to strain-specific samples from uninfected animals and then tolerant and resistant group means were compared to identify differential expression across these phenotypes. Data were normalized to the tolerant group, so a positive value indicates upregulation of a given gene in the resistant group and a negative value indicates upregulation in the tolerant group. Only genes that were at least 2-fold differently expressed across these groups and statistically significantly different (*P* < 0.05) were included (552 genes). These genes were mapped to the QTL regions we identified in the previous section.

*Scq1*, the QTL region linked to STm spleen colonization that we identified on Chr 3, contains 533 genes. Five genes from this region were differentially expressed: fibrinogen alpha chain (*Fga*), fibrinogen beta chain (*Fgb*), fibrinogen gamma chain (*Fgg*), S100 calcium binding protein A5 (*S100a5*), and trichohyalin (*Tchh*) (Table 2). *Fga*, *Fgb*, *Fgg*, and *Tchh* were all upregulated in resistant strains, while *S100a5* was upregulated in tolerant strains. *Fga*, *Fgb*, and *Fgg* are the three subunits of fibrinogen and are involved in clot formation, wound healing, and microbial resistance and are thus strong candidates for further study (42). *S100a5* is expressed primarily in neuronal bodies (43), and *Tchh* is expressed in the tongue and hair (44); neither appears to be involved in the response to infection. Of the 20 genes found in the QTL linked to liver colonization on Chr 13, none were differentially expressed in the spleens of tolerant versus resistant mice.

**TABLE 2:** QTL associations that contain differentially expressed genes in STm tolerant versus STm resistant CC mice^*a*^

QTL region	Gene	Fold change (log_2_)
*Scq1*	*Fga*	7.04
T Chr 6	*Clec2h*	–6.07
*Scq1*	*Fgb*	5.69
*Scq1*	*Fgg*	4.92
T Chr 6	*Clec2j*	–4.27
T Chr 2	*Wfdc13*	–4.07
T Chr 2	*Wfdc10*	–3.75
R Chr 17	*Gm23947*	–3.50
T Chr 6	*Slco1b2*	3.44
T Chr 6	*Gm19434*	–3.43
T Chr 6	*Pzp*	3.34
T Chr 6	*Grin2b*	–2.93
T Chr 6	*Gm26826*	–2.63
T Chr 6	*Iqsec3*	–2.48
T Chr 6	*Mug1*	2.43
R Chr 3	*Spag17*	2.28
T Chr 6	*Gm30524*	–2.14
T Chr 6	*Clec4e*	2.14
*Scq1*	*Tchh*	2.13
R Chr 17	*Gm22346*	2.09
T Chr 6	*Slco1a5*	–2.03
*Scq1*	*S100a5*	–2.02

a Tolerance was used as the baseline in RNA comparison, so the log_2_-fold changes that are positive indicate upregulated in resistant mice and the values that negative indicated upregulated in tolerant mice.

One gene of the 212 genes in the QTL region on Chr 3 associated with spleen colonization (sperm-associated antigen 17 [*Spag17*]) and 2 predicted genes of the 64 genes in the QTL region on Chr 17 associated with liver colonization (*Gm22346* and *Gm23947*) were differentially expressed for the resistance phenotype (Table 2). *Spag17* and *Gm22346* were upregulated in resistant strains, while *Gm23947* was upregulated in tolerant strains. *Spag17* is involved in motility of axonemes, such as sperm flagella and the cilia of the lung epithelium (45). The two predicted genes linked to resistance are of unknown function. The QTL linked to resistance to STm infection on Chr 13 overlaps with the QTL associated with liver colonization on Chr 13 and does not contain any differentially expressed genes.

Of the genes located in the suggestive QTL on Chr 2 associated with the tolerance phenotype, two genes were differentially expressed: WAP four-disulfide core domain 10 (*Wfdc10*) and WAP four-disulfide core domain 13 (*Wfdc13*) (Table 2). These genes have antimicrobial functions and are upregulated in response to exposure to LPS (46). On the suggestive QTL on Chr 6 associated with tolerance, three predicted genes and nine genes were differentially expressed (Table 2). C-type lectin domain family 2, member h (*Clec2h*); C-type lectin domain family 2, member J (*Clec2j*); and C-type lectin domain family 4, member e (*Clec4e*) are members of the same family and are involved in immunity. *Clec2h* and *Clec2j* are upregulated in tolerant strains, while *Clec4e* is upregulated in resistant strains. *Clec2h* is expressed in the intestines and is involved in intestinal monitoring (47, 48), while *Clec4e* is involved in macrophage functioning, as well as cytokine induction (49). *Clec2j* function is unknown (50). PZP, α-2-macroglobulin-like (*Pzp*) is an immunosuppressive protein often expressed during pregnancy and high levels are linked with poor outcomes to infection (51, 52). *Pzp* is upregulated in resistant strains. Solute carrier organic anion transporter family, members 1a5 (*Slco1a5*) and 1b2 (*Slco1b2*) are in the same family. *Slco1a5* and *Slco1b2* are involved in bile acid and bile salt transport, and their deletion leads to a buildup of bilirubin in the blood (53, 54). These genes play a key role in drug uptake, specifically in the liver and intestines. *Slco1a5* is upregulated in tolerant strains, while *Slco1b2* is upregulated in resistant strains.

### Ingenuity pathway analysis revealed pathways involved in response to infection.

RNA sequencing data from both uninfected and infected spleen samples identified putative pathways involved in the response to STm infection. Ingenuity pathway analysis (IPA) identified the top canonical pathways (Table 3).

**TABLE 3:** Top canonical pathways for spleen RNA-seq comparing tolerant and resistant mice at 3 weeks postinfection identified using ingenuity pathway analysis^*a*^

Ingenuity canonical pathway	–Log (*P*)	Ratio	z-score	Proteins
Acute-phase response signaling	15.00	0.11	1.39	ALB, AMBP, APCS, APOA1, APOA2, APOH, C9, CRP, **FGA**, **FGB**, **FGG**, HPX, IL36G, MBL2, PLG, SAA1, SAA2-SAA4, Saa3, SERPINA3, TTR
LXR/RXR activation	14.70	0.14	2.67	ALB, AMBP, APOA1, APOA2, APOB, APOH, C9, **FGA**, GC, HPX, IL1R2, IL1RAPL1, IL36G, KNG1, NOS2, SAA1, TTR
FXR/RXR activation	13.20	0.13	NaN	ALB, AMBP, APOA1, APOA2, APOB, APOH, C9, FBP1, **FGA**, GC, HPX, IL36G, KNG1, SAA1, SLCO1B3, TTR
Xenobiotic metabolism PXR signaling pathway	7.43	0.07	1.94	CES3, CES5A, CHST4, CYP2B6, CYP2C8, CYP3A5, ESD, NOS2, SULT4A1, UGT1A9 (includes others), UGT2B17, UGT2B28, UGT2B7
LPS/IL-1-mediated inhibition of RXR function	6.05	0.05	−1.13	CHST4, Cyp2a12/Cyp2a22, CYP2B6, CYP2C8, Cyp2d9 (includes others), Cyp2j5, CYP3A5, IL1R2, IL1RAPL1, IL36G, SLCO1A2, SLCO1B3, SULT4A1

a The top five pathways are listed. Genes found in QTL regions are highlighted in boldface. NaN, a z-score cannot be assigned because of the lack of data or because the known data conflict.

The five pathways with the highest log score were acute-phase response signaling, LXR/RXR activation, FXR/RXR activation, xenobiotic metabolism PXR signaling pathway, and LPS/IL-1-mediated inhibition of RXR function (Table 3). Acute-phase response signaling, LXR/RXR activation, and xenobiotic metabolism PXR signaling pathway were upregulated in resistant mice. LPS/IL-1-mediated inhibition of RXR function was upregulated in tolerant mice. Acute-phase response signaling, LXR/RXR activation, and FXR/RXR activation all contained the gene *Fga*, and acute-phase response signaling contained the genes *Fgb* and *Fgg*. *Fga*, *Fgb*, and *Fgg* were also found in the QTL regions associated with spleen colonization, suggesting that these genes are the most likely to differentiate between resistance and tolerance.

## DISCUSSION

A variety of host immunologic, physiologic, and metabolic factors likely contribute to differences in disease outcome after exposure to an infectious agent. In previous work, we described our observations around susceptibility and survival after acute *S.* Typhimurium infection in genetically diverse mice (37). However, we found that infection periods longer than 7 days were required to differentiate tolerant and resistant phenotypes because bacterial burden and survival were similar between these strains at 1 week postinfection (31, 32, 37). To differentiate these host disease phenotypes, we infected 18 strains of CC mice, known to survive acute infection, with STm for up to 3 weeks (37).

Seven of these 18 CC strains did not survive 3 weeks postinfection and thus had a survival phenotype we called “delayed susceptible.” Six of the remaining strains fit the criteria for tolerance, surviving infection with a bacterial load that caused serious systemic infection in delayed susceptible strains. Resistant strains were colonized at very low levels compared to their tolerant counterparts and exhibited no clinical signs of disease. When all resistant strains were grouped, they had median colonizations of 1.26 × 10^2^ CFU/g in the liver and 2.82 × 10^3^ CFU/g in the spleen at 3 weeks, which is significantly less than tolerant strains that had medians of 1.29 × 10^4^ CFU/g in the liver and 1.13 × 10^5^ CFU/g in the spleen (see Fig. S6; both *P* < 0.0001).

PP and MLN colonization varied across the CC strains was examined. Long-term STm colonization in mice is linked to the persistence of infection in the reticuloendothelial system, particularly in the MLN (55). In our study, tolerant strains, defined by persistently higher bacterial load, had significantly more STm in their MLN than did resistant strains, supporting a role for the RE system in long-term colonization.

We previously showed that preinfection baseline core body temperature differs between strains that survived or were susceptible to acute STm infections. Surviving strains had cooler preinfection baseline temperatures by ~0.3°C across their resting periods, active periods, and full 24-h periods (37). This difference did not appear to be related to higher or lower locomotor activity (37) but could be the result of differences in metabolism. In our current work, tolerant strains had the lowest baseline core body temperatures across all three time periods compared to resistant and delayed susceptible strains. Tolerant strains also had cooler body temperatures compared to strains susceptible to acute infection by ~0.5°C.

Disruption of circadian patterns of body temperature and activity have previously been reported during parasitic and viral infections (56, 57). We recently reported that circadian patterns of body temperature and activity are disrupted in CC mice acutely infected with STm (37), and this was also true during the longer postinfection periods described here. After infection, the time to disrupted circadian patterns for core body temperature was different between resistant and tolerant strains. Diurnal patterns of core body temperature were disrupted earlier in strains that were tolerant of STm infection than in resistant strains. Thus, in tolerant strains that survive STm infection despite showing signs of disease, we can see earlier symptoms with telemetry that are not otherwise readily observable (57, 58). Lipopolysaccharide (LPS) injection in rats is correlated with suppression of biological clock genes (59). Although LPS may contribute to the circadian pattern disruption in our experiments, different onset and the timing of pattern disruption in mice with different genetic backgrounds suggest that this disruption is more complex than previously appreciated.

Circadian patterns of locomotor activity in infected mice were disrupted later than patterns of core body temperature in all groups of mice (Fig. 3B), although the time to disruption did not vary between STm tolerant and resistant CC mice. Early disruption of body temperature rhythms versus other clinical signs has also been observed in SIV-infected monkeys (57). Detection of circadian pattern disruption was also compared between temperature and activity for each phenotypic group. Disruption of temperature patterns was a more sensitive predictor of disease onset for tolerant and delayed susceptible strains but not for resistant strains.

Differences in the immune response were observable between tolerant and resistant strains. Tolerant strains had a larger rise in white blood cell count driven by a significantly higher increase in neutrophils than resistant strains after infection with STm. Neutrophils are part of the innate immune system and are vital to early control of infections (10). Tolerant animals appear to maintain neutrophilia secondary to prolonged infection with STm. Since resistant strains appear to be clearing the infection, the lower bacterial count may reduce the need for large numbers of neutrophils. Histopathology showed large numbers of neutrophil infiltration in both spleen and liver primarily in tolerant mice, suggesting that in this setting STm is perhaps able to promote and exploit host mediated inflammation for persistence at a systemic site, much as occurs in the intestine (7, 60).

We discovered three cytokines that were significantly different between tolerant and resistant strains. IFN-γ was increased in both resistant and tolerant strains, but tolerant strains had a much larger increase after infection. IFN-γ keeps STm at a manageable level for the host, and neutralizing IFN-γ results in more rapid replication of STm increasing the severity of infection (55). Since tolerant strains have such a high level of IFN-γ, this likely contributes to controlling their bacterial burden and perhaps their disease symptoms.

MCP-1 (CCL-2) is also elevated in STm tolerant mice relative to their resistant counterparts. This CC chemokine is a chemoattractant for monocytes, macrophages, and also for lymphocytes, NK cells, basophils, and dendritic cells (61) and is produced by many cell types. Septic human patients also have elevated serum MCP-1 (62), which can be induced during inflammation by cytokines and chemokines including IFN-γ and other substances such as LPS (63). High levels of MCP-1 have been linked to more severe organ damage/failure, higher levels of septic shock, and higher mortality (64, 65). Transgenic mice that overexpress MCP-1 are more highly susceptible to infections with intracellular bacterial pathogens (63, 66). In models with reduced serum MCP-1, these animals are resistant to infection with Listeria monocytogenes (66). High serum levels of MCP-1 may blind cellular responses to local levels of MCP-1 (63, 66). Finally, polymorphisms in the CCL2 gene have been shown to influence the outcomes of infection with Mycobacterium tuberculosis (67). The MCP-1 elevation in our STm tolerant and resistant animals are consistent with these findings, and yet the tolerant animals are able to survive the increased bacterial load that appears to occur with elevated MCP-1. Since STm resides and replicates in monocytes, high levels of MCP-1 may not be protective (68).

Finally, ENA-78, decreased in tolerant mice and increased in resistant mice, is a neutrophil chemoattractant (69). Tolerant strains have a significantly higher number of circulating neutrophils than resistant strains, despite having less circulating ENA-78. Hepatocytes secrete ENA-78 in response to bacterial invasion or tissue damage (70). Hepatocytes in tolerant strains may be creating the proper chemokine gradients that would attract an influx of neutrophils into the liver, while resistant strains that have started clearing bacteria and repairing damage no longer need an influx of neutrophils (41, 70). Further experiments are required to confirm the role of this cytokine.

Tolerance to infectious agents has been hypothesized to correlate with reduced damage in infected tissues (33). In our experiments, tolerant strains had milder tissue damage than strains that were susceptible to acute STm infection only in spleen at 7 days postinfection (see Fig. S4) (37). However, strains that tolerated STm infection had equivalent tissue damage at 1 and 3 weeks postinfection, showing no evidence of repair (Fig. 6), whereas resistant mice appeared to be actively repairing tissue damage by the 3-week time point. Furthermore, tolerant strains, which were colonized with STm at similar levels to delayed susceptible strains, had tissue damage that was as severe as tissue damage in DS strains. Tissue damage and repair have been linked to tolerance for other pathogens, and yet we did not observe this (71, 72). Our findings suggest that in our system, tissue damage and repair do not appear to distinguish tolerance from other phenotypes. Tolerance to STm infections likely relies on other physiologic, immunologic, and metabolic mechanisms (30, 72).

We used QTL analysis to identify genetic regions associated with resistance, tolerance, and in STm colonization of the spleen and liver. We identified one significant association with spleen colonization, multiple associations with resistance and tolerance, and one suggestive association with liver colonization. However, the small number of CC strains used and the lack of normal distribution of data did not allow us to pinpoint individual genes responsible for these associations (73). RNA-seq on infected spleen allowed us to identify genes that are differentially expressed in tolerant versus resistant strains in response to STm infection, and to identify where these genes overlap our QTL regions. Fourteen of the 22 genes that were differentially expressed between tolerant and resistant strains that are located in QTL regions were associated with tolerance. For the suggestive association with tolerance found on Chr 6, C-type lectin domain family 2 member H (*Clec2h*) was the most highly differentially expressed gene between tolerant and resistant strains. *Clec2h* (also known as C-type lectin-related [*Clr-f*]) is expressed in the ileum, kidney, and liver and in IL-2-activated natural killer cells (50, 74) and plays a role in immune surveillance of the intestinal lumen (48). We show that *Clec2h* is also expressed in spleen. *Clec2h* may facilitate bacterial detection in the spleens in tolerant mice, and thus helping them mount an appropriate immune response to STm. Alternatively, detection of *Clec2h* could result from a large influx of natural killer cells to the spleen in response to the presence of STm. Further work may identify the cell types expressing *Clec2h* during STm infection.

*Fga*, *Fgb*, and *Fgg* are three of the most highly upregulated genes in the spleens of resistant mice and are associated with reduced spleen colonization and the QTL on Chr 3, *Scq1*. Fibrinogen is important in countering initial bacterial colonization by forming a matrix that traps bacteria and by recruiting and activating immune cells (42). Fibrinogen kinetics (formation and degradation) are also vital to wound healing; our histopathology analysis suggests the presence of microabscesses in both spleen and liver that require healing (42). Fibrinogen is also important in bacterial defense. Several bacterial species successfully modify fibrinogen to improve their proliferation, particularly S. aureus, supporting an evolutionary battle between fibrinogen and bacteria (75). STm infections can alter kinetics of thrombus formation (clots) in the spleen and liver, further supporting an interaction between fibrinogen and STm (76). In our work, the fibrinogen genes were some of the most highly differentially expressed genes, suggesting that high fibrinogen expression is a previously unknown resistance mechanism.

We had previously defined and characterized a group of collaborative cross strains that are highly susceptible to acute STm infection and identified several regions of the mouse genome involved (37). In the present study, we identified multiple phenotypes in response to long-term STm infection—delayed susceptibility, tolerance, and resistance—and characterized several hallmarks of these phenotypes. STm tolerant CC strains have lower core body temperature preinfection and higher, more persistent bacterial colonization than resistant strains with minimal clinical signs of disease. Disruption of the core body temperature diurnal rhythm of tolerant animals is a very early sign of disease, while gross disruptions in locomotor activity occur with a >24-h lag. During long-term infection, tolerant strains develop and do not repair damage in the spleen and liver, maintain neutrophilia, have elevated levels of circulating MCP-1 and IFN-γ, and show a reduction in ENA-78 relative to resistant strains. High expression of *Clec2h*, located on the QTL on Chr 6, is correlated with tolerance, and the mechanistic basis underlying this association remains to be explored. Finally, we show that multiple fibrinogen genes on the QTL *Scq1* on Chr 3 are highly expressed in resistant strains, potentially revealing a new pathway involved in resistance to Salmonella infection. While host factors involved in host-pathogen interactions in this work may define the phenotypes we have described here, nongenetic factors (microbiota, for example) could contribute to some of the phenotypes we observed. This idea will be explored in future work.

## MATERIALS AND METHODS

### Bacterial strains and media.

The Salmonella enterica serovar Typhimurium strain (HA420) used for this study was derived from ATCC 14028. HA420 is a fully virulent, spontaneous nalidixic acid-resistant derivative of ATCC 14028 (77). Strains were routinely cultured in Luria-Bertani (LB) broth and plates, supplemented with antibiotics when needed at 50 mg/L nalidixic acid (Nal).

For murine infections, strains were grown aerobically at 37°C to stationary phase in LB broth with nalidixic acid and diluted to generate an inoculum of 2 × 10^7^ to 5 × 10^7^ organisms in 100 μL. Bacterial cultures used as inoculum were serially diluted and plated to enumerate CFU to determine the exact titer.

### Murine strains.

Collaborative-cross (CC) mice were used in these experiments. All CC strains were obtained from UNC’s Systems Genetics Core Facility (SGCF) and either used directly for experiments or subsequently bred independently at the Division of Comparative Medicine, Texas A&M University, prior to these experiments. Our experiments utilized 18 strains of CC mice, with 3 females and 3 males per strain, for a total of 112 mice (CC045 only had 2 females due to an experimental complication) (see Table S4). Mice were fed Envigo Teklad Global 19% protein extruded rodent diet (irradiated, 2919) or Envigo Teklad rodent diet (8604) based on strain need.

### Ethics statement.

All animal experiments were conducted in accordance with the *Guide for the Care and Use of Laboratory Animals* and with the approval of the TAMU Institutional Animal Care and Use Committee (IACUC) under animal use protocol numbers: AUP 2018-0488 D and 2015-0315 D.

### Placement of telemetry devices.

Five- to nine-week-old CC mice were anesthetized with isoflurane anesthesia. The abdomen was opened with a midline abdominal incision (up to 2 cm). Starr Life Sciences G2 E-mitter devices were loosely sutured to the ventral abdominal wall as previously described (37) to continuously monitor core body temperature and activity. Implanted mice were group housed and monitored twice daily for signs of pain and to ensure wound closure for 7 days postsurgery. Any animals found to have serious complications after surgery were humanely euthanized. Clips were removed at 7 days postsurgery.

### Infection with *Salmonella* Typhimurium.

After 7 days of acclimation in the BSL-2 facility, 8- to 12-week-old implanted CC mice were weighed and infected by gavage with a dose of 2 × 10^7^ to 5 × 10^7^ CFU of *S.* Typhimurium HA420 in 100 μL of LB broth as previously described (37). Infected mice were monitored twice daily for signs of disease and activity by visual inspection. When telemetry data and health condition data suggested the development of clinical disease from infection, mice were humanely euthanized. If animals remained clinically healthy throughout the duration of the experiment, they were humanely euthanized at 21 days postinfection.

### Bacterial load determination.

Mice were humanely euthanized by CO_2_ asphyxiation, and the spleen, liver, ileum, cecum, colon, Peyer’s patches, and MLN were collected. A third of each organ was collected in 3 mL of ice-cold phosphate-buffered saline (PBS), weighed, homogenized, serially diluted in 1× PBS, and plated on Nal plates for enumeration of *S*. Typhimurium in each organ. Peyer’s patches and MLN were collected whole. The data are expressed as CFU/g of tissue.

### Telemetry monitoring.

Prior to placing implanted mice on telemetry platforms, mice were moved into individual cages and provided with a cardboard hut and bedding material. Individual cages containing implanted, uninfected mice were placed onto ER4000 receiver platforms, and the collection of body temperature (once per minute) and gross motor activity (continuous measurement summed each minute) data was initiated. Body temperature and gross motor activity data were collected for 7 days from uninfected mice. Mice were removed briefly from the receiver platforms for infection and then placed back on the platforms, and data collection was resumed. Infected animals were continuously monitored by telemetry in addition to twice daily visual monitoring.

### Identification of deviation from circadian pattern of body temperature.

Additional clinical information, such as the time of inoculation relative to the start of the experiment (denoted “T”), was used in centering time series for comparison between mice (typically, 7 days after the beginning of monitoring).

Quantitative detection of deviation from the baseline “off-pattern,” using temperature data were calculated on an individual basis. A temperature time series was filtered, a definition of healthy variation was defined, and then the time of first “off-pattern” was calculated using that definition on postinoculation data.

Each mouse time series was preprocessed using a moving median filter with a 1-day window. For a specific minute *t*, the median collection of temperature values from [*t*–720, *t*+720] was used in calculating a median for the value *t*. After this processing, healthy variation was defined as any temperature falling within the range of minimum to maximum values during the preinoculation phase [T–5760,T] (5,760 min = 4 days). This choice allowed for enough data to account for natural interday variation due to potential factors such as interstrain variation, epigenetic differences, and sex differences, while avoiding bias due to observed acclimation time after transfer to a new facility in some mice in the first few days of observation (see Fig. S5A).

10.1128/mbio.01120-22.5 FIG S5 Examples of telemetry calculations. Illustrations of how temperature (A) and activity (B) calculations are shown. Specifically, the maximum, median, and minimum preinfection temperatures and activities, as well the “off-patterns” for postinfection, are shown. Download FIG S5, TIF file, 2.5 MB. Copyright © 2022 Scoggin et al. 2022 Scoggin et al. https://creativecommons.org/licenses/by/4.0/ This content is distributed under the terms of the Creative Commons Attribution 4.0 International license.

Identifying postinoculation off-pattern behavior was done by identifying temperature values that fall outside the interval of healthy variation. The postinoculation interval ranges from [T+60,T+30240] (21 days), where the 1 h gap was used to avoid false-positive detection due to the physical disturbance associated with inoculation (see Fig. S5A).

### Detection of deviation from circadian pattern of activity.

While activity data do exhibit circadian patterns, these data necessitated a different approach to preprocessing compared to temperature data because activity values are inherently nonnegative and have a modal value of 0. Two approaches were taken. One was from the perspective of parameter estimation of independent and identically distributed sampling from a statistical distribution. The second approach was based on plainly calculating the fraction of nonzero activity values measured in a moving window. Hence, we approached the analysis of activity data from the perspective of determining the parameters of a stochastic process. In the first approach, for a given time interval *t*, we worked from the assumption that the number of activity values observed to be *i* obeys the following distribution:

ln⁡(p(A(t)=i))=α+βi

where the coefficient β, expected to be negative, corresponds to the modeling assumption that the relative drop in observed activity counts ought to decay (note 0 < e^β < 1 if β is negative). For instance, if the value of β = −0.693, so that e^β^ ≈ 1\/2, this would represent an assumption that there are half as many activity values observed to be 1 (one movement per minute) than 2 (two movements per minute). In actuality, this decay coefficient is typically seen to be β ≈ –0.025 to β ≈ –0.015, representing that observed activity values decay by half around every 27 to 46 values.

This theory is implemented in practice by windowing a mouse’s activity time series, creating a binning (empirical distribution) [*i*, *c*(*i*)], the performing a log-linear fit by transforming *c*(*i*) → ln[1 + *c*(*i*)] and applying linear least-squares regression to calculate α and β. Statistical models with simpler assumptions stemming from a “memoryless” assumption were attempted but did not yield any feasible agreement with the observed activity data (see Fig. S5B).

The second approach mentioned above produced a qualitatively cleaner signal, which again is based on windowing a mouse’s activity time series and calculating the fraction of activity values in a window for which *A*(*t*) > 0. One might expect not weighting by intensity of activity loses information, but we saw this approach to filtering activity data resulted in a time series for which we could apply the same methods as with the temperature time series (see Fig. S5B).

### Histopathology.

After euthanasia, liver, spleen, ileum, cecum, and colon samples from each mouse were collected and fixed in 10% neutral buffered formalin at room temperature for 24 h, stored in 70% ethanol before embedding in paraffin, sectioned at 5 μm, and stained with H&E. Histologic sections of all tissues were evaluated in a blinded manner by bright field microscopy and scored on a scale from 0 to 4 for tissue damage by a board-certified veterinary pathologist (see Table S3). The combined scores of spleens and livers from individual mice were used to calculate the medians and interquartile ranges for each group. Whole slide images of liver and spleen H&E-stained sections were captured as digital files by scanning at 40× using a 3DHistech Pannoramic Scan II FL scanner (Epredia, Kalamazoo, MI). Digital files were processed by Aiforia Hub (Aiforia, Cambridge, MA) software for generating the images with 100-μm scale bars.

### Complete blood count.

Whole blood was collected by cheek bleed for a complete blood count (CBC) 1 week prior to surgery for the preinfection sample. Blood from infected animals was collected at necropsy by cardiac puncture. Blood was collected from additional uninfected control animals by cardiac puncture, and median values were used as the baseline for mice that did not serve as their own preinfection control. All blood was collected into EDTA tubes and analyzed on an Abaxis VetScan HM5.

### Cytokine and chemokine analysis.

Serum was stored at –80°C and thawed on ice immediately prior to cytokine assays. Serum cytokine levels were evaluated using an Invitrogen ProcaratPlex Cytokine/Chemokine Convenience Panel 1A 36-plex kit according to the manufacturer’s instructions (Thermo Fisher). Briefly, magnetic beads were added to each well of the 96-well plate and washed on the Bio-Plex Pro wash station. Then, 25-μL samples and standards were added to the plate, along with 25 μL of universal assay buffer. Plates were shaken at room temperature for 1 h and washed. Next, 25 μL of detection antibody was added, followed by incubation for 30 min and then washed away, followed in turn by 50 μL of streptavidin-PE for 30 min and washed away. Then, 120 μL of reading buffer was added, and the plates were evaluated using a Bio-Plex 200 (Bio-Rad). Samples and standards were assayed in duplicate, and samples were diluted as needed to get 25 μL of serum per duplicate. The kit screens for the following 36 cytokines and chemokines: IFN-γ, IL-12p70, IL-13, IL-1β, IL-2, IL-4, IL-5, IL-6, TNF-α, GM-CSF, IL-18, IL-10, IL-17A, IL-22, IL-23, IL-27, IL-9, GROα, IP-10, MCP-1, MCP-3, MIP-1α, MIP-1β, MIP-2, RANTES, eotaxins, IFN-α, IL-15/IL-15R, IL-28, IL-31, IL-1α, IL-3, G-CSF, LIF, ENA-78/CXCL5, and M-CSF. Median values of uninfected animals for each strain were used as the baseline for that strain.

### QTL analysis.

gQTL, an online resource designed specifically to identify CC QTLs, was used to find putative QTL associations (78). Briefly, median values of each strain for various parameters were uploaded to the website, and QTL analysis was performed using 1,000 permutations with “automatic” transformation. Automatic transformation picks either log or square root transformations, whichever best normalizes the data.

### Transcriptomic analysis.

Spleen tissue was collected from all animals of all lines at necropsy, snap-frozen in liquid nitrogen, and stored in a –80°C freezer until RNA-seq could be performed. All molecular work was performed in the Molecular Genomics Core of the Texas A&M Institute for Genome Sciences and Society (TIGSS). The following protocol was adapted from the Molecular Genomics Core, and TIGSS personnel aided in data acquisition and plot generation. Spleens were homogenized in TRIzol. RNA samples were quantified with a Qubit Fluorometer (Life Technologies) with a broad-range RNA assay, and concentrations were normalized for library preparation. RNA quality was verified on an Agilent TapeStation with a broad range RNA ScreenTape. Total RNA sequencing libraries were prepared using the Illumina TruSeq stranded mRNA-seq preparation kit. Barcoded libraries were pooled at equimolar concentrations and sequenced on an Illumina NovaSeq 6000 2 × 150 S4 flow cell. RNA-seq libraries were trimmed to remove adapter sequences and low-quality bases using TrimGalore version 0.6.6, with Cutadapt version 3.0 and FastQC version 0.11.9.

The Colorado State University team performed data processing of the RNA-seq using a pipeline consisting of STAR alignment (79), FeatureCounts (80), and then DeSeq2 R package was used for differential gene expression analysis (81). The STAR alignment and FeatureCounts were done using mouse genome version GRCm38 Ensembl version 100 available from Ensembl (82). All infected samples were normalized to their uninfected control samples (or an average uninfected control if no strain specific samples were available). These strain values were then grouped by infection response and a tolerant or resistant mean was used for comparisons between the groups. Tolerant was then set as the baseline, so genes with positive values were upregulated in resistant strains and downregulated in tolerant strains, while genes with negative values were downregulated in resistant strains and upregulated in tolerant strains.

## References

[1] Roy MF, Malo D. 2002. Genetic regulation of host responses to *Salmonella* infection in mice. Genes Immun 3:381–393. doi:10.1038/sj.gene.6363924. 12424619

[2] Santos RL, Raffatellu M, Bevins CL, Adams LG, Tükel C, Tsolis RM, Bäumler AJ. 2009. Life in the inflamed intestine, *Salmonella* style. Trends Microbiol 17:498–506. doi:10.1016/j.tim.2009.08.008. 19819699 PMC3235402

[3] Majowicz SE, Musto J, Scallan E, Angulo FJ, Kirk M, O’Brien SJ, Jones TF, Fazil A, Hoekstra RM, International Collaboration on Enteric Disease “Burden of Illness” Studies. 2010. The global burden of nontyphoidal *Salmonella* gastroenteritis. Clin Infect Dis 50:882–889. doi:10.1086/650733. 20158401

[4] Gilchrist JJ, MacLennan CA, Hill AVS. 2015. Genetic susceptibility to invasive *Salmonella* disease. Nat Rev Immunol 15:452–463. doi:10.1038/nri3858. 26109132

[5] Stanaway JD, Parisi A, Sarkar K, Blacker BF, Reiner RC, Hay SI, Nixon MR, Dolecek C, James SL, Mokdad AH, Abebe G, Ahmadian E, Alahdab F, Alemnew BTT, Alipour V, Allah Bakeshei F, Animut MD, Ansari F, Arabloo J, Asfaw ET, Bagherzadeh M, Bassat Q, Belayneh YMM, Carvalho F, Daryani A, Demeke FM, Demis ABB, Dubey M, Duken EE, Dunachie SJ, et al. 2019. The global burden of non-typhoidal *Salmonella* invasive disease: a systematic analysis for the Global Burden of Disease Study 2017. Lancet Infect Dis 19:1312–1324. doi:10.1016/S1473-3099(19)30418-9. 31562022 PMC6892270

[6] Gordon MA. 2008. *Salmonella* infections in immunocompromised adults. J Infect 56:413–422. doi:10.1016/j.jinf.2008.03.012. 18474400

[7] Keestra-Gounder AM, Tsolis RM, Bäumler AJ. 2015. Now you see me, now you don’t: the interaction of *Salmonella* with innate immune receptors. Nat Rev Microbiol 13:206–216. doi:10.1038/nrmicro3428. 25749454

[8] Lalmanach AC, Montagne A, Menanteau P, Lantier F. 2001. Effect of the mouse Nramp1 genotype on the expression of IFN-γ gene in early response to *Salmonella* infection. Microbes Infect 3:639–644. doi:10.1016/S1286-4579(01)01419-8. 11445450

[9] Arpaia N, Godec J, Lau L, Sivick KE, McLaughlin LM, Jones MB, Dracheva T, Peterson SN, Monack DM, Barton GM. 2011. TLR signaling is required for *Salmonella* Typhimurium virulence. Cell 144:675–688. doi:10.1016/j.cell.2011.01.031. 21376231 PMC3063366

[10] Mastroeni P, Ugrinovic S, Chandra A, MacLennan C, Doffinger R, Kumararatne D. 2003. Resistance and susceptibility to *Salmonella* infections: lessons from mice and patients with immunodeficiencies. Rev Med Microbiol 14:53–62. doi:10.1097/00013542-200304000-00002.

[11] Caron J, Loredo-Osti JC, Laroche L, Skamene E, Morgan K, Malo D. 2002. Identification of genetic loci controlling bacterial clearance in experimental *Salmonella* Enteritidis infection: an unexpected role of Nramp1 (Slc11a1) in the persistence of infection in mice. Genes Immun 3:196–204. doi:10.1038/sj.gene.6363850. 12058254

[12] Sancho-Shimizu V, Malo D. 2006. Sequencing, expression, and functional analyses support the candidacy of Ncf2 in susceptibility to *Salmonella* Typhimurium infection in wild-derived mice. J Immunol 176:6954–6961. doi:10.4049/jimmunol.176.11.6954. 16709856

[13] Khan RT, Chevenon M, Yuki KE, Malo D. 2014. Genetic dissection of the Ity3 locus identifies a role for Ncf2 co-expression modules and suggests Selp as a candidate gene underlying the Ity3.2 locus. Front Immunol 5:375. doi:10.3389/fimmu.2014.00375. 25161653 PMC4129629

[14] Li Q, Cherayil BJ. 2003. Role of Toll-like receptor 4 in macrophage activation and tolerance during *Salmonella enterica* serovar Typhimurium infection. Infect Immun 71:4873–4882. doi:10.1128/IAI.71.9.4873-4882.2003. 12933828 PMC187311

[15] Bihl F, Larivière L, Qureshi ST, Flaherty L, Malo D. 2001. LPS-hyporesponsiveness of *mnd* mice is associated with a mutation in Toll-like receptor 4. Genes Immun 2:56–59. doi:10.1038/sj.gene.6363732. 11294571

[16] Churchill GA, Airey DC, Allayee H, et al. 2004. The collaborative cross, a community resource for the genetic analysis of complex traits. Nat Genet 36:1133–1137. doi:10.1038/ng1104-1133. 15514660

[17] Aylor DL, Valdar W, Foulds-Mathes W, Buus RJ, Verdugo RA, Baric RS, Ferris MT, Frelinger JA, Heise M, Frieman MB, Gralinski LE, Bell TA, Didion JD, Hua K, Nehrenberg DL, Powell CL, Steigerwalt J, Xie Y, Kelada SNP, Collins FS, Yang IV, Schwartz DA, Branstetter LA, Chesler EJ, Miller DR, Spence J, Liu EY, McMillan L, Sarkar A, Wang J, Wang W, Zhang Q, Broman KW, Korstanje R, Durrant C, Mott R, Iraqi FA, Pomp D, Threadgill D, de Villena FP-M, Churchill GA. 2011. Genetic analysis of complex traits in the emerging collaborative genetic analysis of complex traits in the emerging collaborative cross. Genome Res 21:1213–1222. doi:10.1101/gr.111310.110. 21406540 PMC3149489

[18] Threadgill DW, Miller DR, Churchill GA, de Villena FP-M. 2011. The collaborative cross: a recombinant inbred mouse population for the systems genetic era. Ilar J 52:24–31. doi:10.1093/ilar.52.1.24. 21411855

[19] Collaborative Cross Consortium. 2012. The genome architecture of the collaborative cross mouse genetic reference population. Genetics 190:389–401. doi:10.1534/genetics.111.132639. 22345608 PMC3276630

[20] Phillippi J, Xie Y, Miller DR, Bell TA, Zhang Z, Lenarcic AB, Aylor DL, Krovi SH, Threadgill DW, de Villena FP-M, Wang W, Valdar W, Frelinger JA. 2014. Using the emerging collaborative cross to probe the immune system. Genes Immun 15:38–46. doi:10.1038/gene.2013.59. 24195963 PMC4004367

[21] Abu Toamih Atamni H, Nashef A, Iraqi FA. 2018. The collaborative cross mouse model for dissecting genetic susceptibility to infectious diseases. Mamm Genome 29:471–487. doi:10.1007/s00335-018-9768-1. 30143822

[22] Smith CM, Sassetti CM. 2018. Modeling diversity: do homogeneous laboratory strains limit discovery? Trends Microbiol 26:892–895. doi:10.1016/j.tim.2018.08.002. 30166218 PMC6610874

[23] Korth MJ, Tchitchek N, Benecke A, Katze MG. 2013. Systems approach to influenza-virus host interactions and the pathogenesis of highly virulent and pandemic viruses. Semin Immunol 25:228–239. doi:10.1016/j.smim.2012.11.001. 23218769 PMC3596458

[24] Rasmussen AL, Okumura A, Ferris MT, Green R, Feldmann F, Kelly SM, Scott DP, Safronetz D, Haddock E, LaCasse R, Thomas MJ, Sova P, Carter VS, Weiss JM, Miller DR, Shaw GD, Korth MJ, Heise MT, Baric RS, de Villena FP-M, Feldmann H, Katze MG. 2014. Host genetic diversity enables Ebola hemorrhagic fever pathogenesis and resistance. Science 346:987–991. doi:10.1126/science.1259595. 25359852 PMC4241145

[25] Price A, Okumura A, Haddock E, Feldmann F, Meade-White K, Sharma P, Artami M, Lipkin WI, Threadgill DW, Feldmann H, Rasmussen AL. 2020. Transcriptional correlates of tolerance and lethality in mice predict Ebola virus disease patient outcomes. Cell Rep 30:1702–1713. doi:10.1016/j.celrep.2020.01.026. 32049004 PMC11062563

[26] Plant JE, Glynn AA. 1982. Genetic control of resistance to *Salmonella* Typhimurium infection in high and low antibody responder mice. Clin Exp Immunol 50:283–290. 6817955 PMC1536681

[27] Kover PX, Schaal BA. 2002. Genetic variation for disease resistance and tolerance among *Arabidopsis thaliana* accessions. Proc Natl Acad Sci USA 99:11270–11274. doi:10.1073/pnas.102288999. 12172004 PMC123246

[28] Howick VM, Lazzaro BP. 2014. Genotype and diet shape resistance and tolerance across distinct phases of bacterial infection. BMC Evol Biol 14:56–13. doi:10.1186/1471-2148-14-56. 24655914 PMC3997931

[29] Merkling SH, Bronkhorst AW, Kramer JM, Overheul GJ, Schenck A, Van Rij RP. 2015. The epigenetic regulator G9a mediates tolerance to RNA virus infection in *Drosophila*. PLoS Pathog 11:e1004692–25. doi:10.1371/journal.ppat.1004692. 25880195 PMC4399909

[30] Louie A, Song KH, Hotson A, Thomas Tate A, Schneider DS. 2016. How many parameters does it take to describe disease tolerance? PLoS Biol 14:e1002435–21. doi:10.1371/journal.pbio.1002435. 27088212 PMC4835111

[31] Raberg L, Sim D, Read AF. 2007. Disentangling genetic variation for resistance and tolerance to infectious disease in animals. Science 318:812–814. doi:10.1126/science.1148526. 17975068

[32] Read AF, Graham AL, Råberg L. 2008. Animal defenses against infectious agents: is damage control more important than pathogen control? PLoS Biol 6:e1000004–2641. doi:10.1371/journal.pbio.1000004. PMC2605932 19222305

[33] Medzhitov R, Schneider DS, Soares MP. 2012. Disease tolerance as a defense strategy. Science 335:936–941. doi:10.1126/science.1214935. 22363001 PMC3564547

[34] Martins R, Carlos AR, Braza F, Thompson JA, Bastos-Amador P, Ramos S, Soares MP. 2019. Disease tolerance as an inherent component of immunity. Annu Rev Immunol 37:405–437. doi:10.1146/annurev-immunol-042718-041739. 30673535

[35] Ayres JS, Schneider DS. 2008. Two ways to survive an infection: what resistance and tolerance can teach us about treatments for infectious diseases. Nat Rev Immunol 8:889–895. doi:10.1038/nri2432. 18927577 PMC4368196

[36] Malo D, Skamene E. 1994. Genetic control of host resistance to infection. Trends Genet 10:365–371. doi:10.1016/0168-9525(94)90133-3. 7985241

[37] Scoggin K, Lynch R, Gupta J, Nagarajan A, Sheffield M, Elsaadi A, Bowden C, Aminian M, Peterson A, Adams LG, Kirby M, Threadgill DW, Andrews-Polymenis HL. 2022. Genetic background influences survival of infections with *Salmonella enterica* serovar Typhimurium in the collaborative cross. PLoS Genet 18:e1010075. doi:10.1371/journal.pgen.1010075. 35417454 PMC9067680

[38] Deshmane SL, Kremlev S, Amini S, Sawaya BE. 2009. Monocyte chemoattractant protein-1 (MCP-1): an overview. J Interferon Cytokine Res 29:313–325. doi:10.1089/jir.2008.0027. 19441883 PMC2755091

[39] Ingram JP, Brodsky IE, Balachandran S. 2017. Interferon-γ in *Salmonella* pathogenesis: new tricks for an old dog. Cytokine 98:27–32. doi:10.1016/j.cyto.2016.10.009. 27773552 PMC5398957

[40] Koltsova EK, Ley K. 2010. The mysterious ways of the chemokine cxcl5. Immunity 33:7–9. doi:10.1016/j.immuni.2010.07.012. 20643334

[41] Mei J, Liu Y, Dai N, Favara M, Greene T, Jeyaseelan S, Poncz M, Lee JS, Worthen GS. 2010. CXCL5 regulates chemokine scavenging and pulmonary host defense to bacterial infection. Immunity 33:106–117. doi:10.1016/j.immuni.2010.07.009. 20643340 PMC3748840

[42] Kearney KJ, Ariëns RAS, Macrae FL. 2022. The Role of Fibrin(ogen) in Wound Healing and Infection Control. Semin Thromb Hemost 48:174–187. doi:10.1055/s-0041-1732467. 34428799

[43] Ravasi T, Hsu K, Goyette J, Schroder K, Yang Z, Rahimi F, Miranda LP, Alewood PF, Hume DA, Geczy C. 2004. Probing the S100 protein family through genomic and functional analysis. Genomics 84:10–22. doi:10.1016/j.ygeno.2004.02.002. 15203200

[44] O’Keefe EJ, Hamilton EH, Lee SC, Steinertt P. 1993. Trichohyalin: a structural protein of hair, tongue, nail, and epidermis. J Invest Dermatol 101:65S–71S. doi:10.1111/1523-1747.ep12362866. 7686953

[45] Abdelhamed Z, Lukacs M, Cindric S, Ali S, Omran H, Stottmann RW. 2020. A novel hypomorphic allele of Spag17 causes primary ciliary dyskinesia phenotypes in mice. Dis Model Mech 13:dmm045344. doi:10.1242/DMM.048645. 32988999 PMC7648611

[46] Andrade AD, Almeida PGC, Mariani NAP, Freitas GA, Kushima H, Filadelpho AL, Spadella MA, Avellar MCW, Silva EJR. 2021. Lipopolysaccharide-induced epididymitis modifies the transcriptional profile of Wfdc genes in mice. Biol Reprod 104:144–158. doi:10.1093/biolre/ioaa189. 33034631

[47] Rutkowski E, Leibelt S, Born C, Friede ME, Bauer S, Weil S, Koch J, Steinle A. 2017. Clr-a: a novel immune-related C-type lectin-like molecule exclusively expressed by mouse gut epithelium. J Immunol 198:916–926. doi:10.4049/jimmunol.1600666. 27956531

[48] Leibelt S, Friede ME, Rohe C, Gütle D, Rutkowski E, Weigert A, Kveberg L, Vaage JT, Hornef MW, Steinle A. 2015. Dedicated immunosensing of the mouse intestinal epithelium facilitated by a pair of genetically coupled lectin-like receptors. Mucosal Immunol 8:232–242. doi:10.1038/mi.2014.60. 24985083

[49] Pahari S, Negi S, Aqdas M, Arnett E, Schlesinger LS, Agrewala JN. 2020. Induction of autophagy through CLEC4E in combination with TLR4: an innovative strategy to restrict the survival of *Mycobacterium tuberculosis*. Autophagy 16:1021–1043. doi:10.1080/15548627.2019.1658436. 31462144 PMC7469444

[50] Zhang Q, Rahim MMA, Allan DSJ, Tu MM, Belanger S, Abou-Samra E, Ma J, Sekhon HS, Fairhead T, Zein HS, Carlyle JR, Anderson SK, Makrigiannis AP. 2012. Mouse Nkrp1-Clr gene cluster sequence and expression analyses reveal conservation of tissue-specific MHC-independent immunosurveillance. PLoS One 7:e50561. doi:10.1371/journal.pone.0050561. 23226525 PMC3514311

[51] Finch S, Shoemark A, Dicker AJ, Keir HR, Smith A, Ong S, Tan B, Choi J-Y, Fardon TC, Cassidy D, Huang JTJ, Chalmers JD. 2019. Pregnancy zone protein is associated with airway infection, neutrophil extracellular trap formation, and disease severity in bronchiectasis. Am J Respir Crit Care Med 200:992–1001. doi:10.1164/rccm.201812-2351OC. 31264895 PMC6794104

[52] Krause K, Azouz F, Nakano E, Nerurkar VR, Kumar M. 2019. Deletion of pregnancy zone protein and murinoglobulin-1 restricts the pathogenesis of West Nile virus infection in mice. Front Microbiol 10:259–259. doi:10.3389/fmicb.2019.00259. 30814992 PMC6381297

[53] van de Steeg E, Wagenaar E, van der Kruijssen CMM, Burggraaff JEC, de Waart DR, Elferink RPJO, Kenworthy KE, Schinkel AH. 2010. Organic anion transporting polypeptide 1a/1b-knockout mice provide insights into hepatic handling of bilirubin, bile acids, and drugs. J Clin Invest 120:2942–2952. doi:10.1172/JCI42168. 20644253 PMC2912192

[54] Hagenbuch B, Meier PJ. 2004. Organic anion transporting polypeptides of the OATP/SLC21 family: phylogenetic classification as OATP/SLCO super-family, new nomenclature and molecular/functional properties. Pflugers Arch 447:653–665. doi:10.1007/s00424-003-1168-y. 14579113

[55] Monack DM, Bouley DM, Falkow S. 2004. *Salmonella* Typhimurium persists within macrophages in the mesenteric lymph nodes of chronically infected Nramp1^+/+^ mice and can be reactivated by IFN-γ neutralization. J Exp Med 199:231–241. doi:10.1084/jem.20031319. 14734525 PMC2211772

[56] Prior KF, O’Donnell AJ, Rund SSC, Savill NJ, van der Veen DR, Reece SE. 2019. Host circadian rhythms are disrupted during malaria infection in parasite genotype-specific manners. Sci Rep 9:1–12. doi:10.1038/s41598-019-47191-8. 31358780 PMC6662749

[57] Huitron-Resendiz S, Marcondes MCG, Flynn CT, Lanigan CMS, Fox HS. 2007. Effects of simian immunodeficiency virus on the circadian rhythms of body temperature and gross locomotor activity. Proc Natl Acad Sci USA 104:15138–15143. doi:10.1073/pnas.0707171104. 17846423 PMC1986626

[58] Lough G, Kyriazakis I, Bergmann S, Lengeling A, Doeschl-Wilson AB. 2015. Health trajectories reveal the dynamic contributions of host genetic resistance and tolerance to infection outcome. Proc Biol Sci 282:20152151. doi:10.1098/rspb.2015.2151. 26582028 PMC4685823

[59] Okada K, Yano M, Doki Y, Azama T, Iwanaga H, Miki H, Nakayama M, Miyata H, Takiguchi S, Fujiwara Y, Yasuda T, Ishida N, Monden M. 2008. Injection of LPS causes transient suppression of biological clock genes in rats. J Surg Res 145:5–12. doi:10.1016/j.jss.2007.01.010. 18279697

[60] Haraga A, Ohlson MB, Miller SI. 2008. Salmonellae interplay with host cells. Nat Rev Microbiol 6:53–66. doi:10.1038/nrmicro1788. 18026123

[61] Gschwandtner M, Derler R, Midwood KS. 2019. More than just attractive: how CCL2 influences myeloid cell behavior beyond chemotaxis. Front Immunol 10:2759. doi:10.3389/fimmu.2019.02759. 31921102 PMC6923224

[62] Bossink AWJ, Paemen L, Jansen PM, Hack CE, Thijs LG, Van Damme J. 1995. Plasma levels of the chemokines monocyte chemotactic proteins-1 and -2 are elevated in human sepsis. Blood 86:3841–3847. doi:10.1182/blood.V86.10.3841.bloodjournal86103841. 7579352

[63] Van Coillie E, Van Damme J, Opdenakker G. 1999. The MCP/eotaxin subfamily of CC chemokines. Cytokine Growth Factor Rev 10:61–86. doi:10.1016/S1359-6101(99)00005-2. 10379912

[64] Martin GS. 2012. Sepsis, severe sepsis and septic shock: changes in incidence, pathogens and outcomes. Expert Rev Anti Infect Ther 10:701–706. doi:10.1586/eri.12.50. 22734959 PMC3488423

[65] Chaudhry H, Zhou J, Zhong Y, Ali MM, McGuire F, Nagarkatti PS, Nagarkatti M. 2015. Role of cytokines as a double-edged sword in sepsis. In Vivo 27:669–684. PMC4378830 24292568

[66] Rutledge BJ, Rayburn H, Rosenberg R, North RJ, Gladue RP, Corless CL, Rollins BJ. 1995. High level monocyte chemoattractant protein-1 expression in transgenic mice increases their susceptibility to intracellular pathogens. J Immunol 155:4838–4843. 7594486

[67] Feng W-X, Flores-Villanueva PO, Mokrousov I, Wu X-R, Xiao J, Jiao W-W, Sun L, Miao Q, Shen C, Shen D, Liu F, Jia Z-W, Shen A. 2012. CCL2-2518 (A/G) polymorphisms and tuberculosis susceptibility: a meta-analysis. Int J Tuberc Lung Dis 16:150–156. doi:10.5588/ijtld.11.0205. 22137597

[68] Hui WW, Hercik K, Belsare S, Alugubelly N, Clapp B, Rinaldi C, Edelmann MJ. 2018. *Salmonella enterica* serovar Typhimurium alters the extracellular proteome of macrophages and leads to the production of proinflammatory exosomes. Infect Immun 86:e00386-17. doi:10.1128/IAI.00386-17. 29158431 PMC5778363

[69] Eckmann L, Kagnoff MF. 2001. Cytokines in host defense against *Salmonella*. Microbes Infect 3:1191–1200. doi:10.1016/S1286-4579(01)01479-4. 11755407

[70] Rowell DL, Eckmann L, Dwinell MB, Carpenter SP, Raucy JL, Yang SK, Kagnoff MF. 1997. Human hepatocytes express an array of proinflammatory cytokines after agonist stimulation or bacterial invasion. Am J Physiol 273:G322–G332. doi:10.1152/ajpgi.1997.273.2.G322. 9277410

[71] Raberg L, Graham AL, Read AF. 2009. Decomposing health: tolerance and resistance to parasites in animals. Philos Trans R Soc Lond B Biol Sci 364:37–49. doi:10.1098/rstb.2008.0184. 18926971 PMC2666700

[72] McCarville JL, Ayres JS. 2018. Disease tolerance: concept and mechanisms. Curr Opin Immunol 50:88–93. doi:10.1016/j.coi.2017.12.003. 29253642 PMC5884632

[73] Ram R, Mehta M, Balmer L, Gatti DM, Morahan G. 2014. Rapid identification of major-effect genes using the collaborative cross. Genetics 198:75–86. doi:10.1534/genetics.114.163014. 25236450 PMC4174955

[74] Plougastel B, Dubbelde C, Yokoyama WM. 2001. Cloning of Clr, a new family of lectin-like genes localized between mouse Nkrp1a and Cd69. Immunogenetics 53:209–214. doi:10.1007/s002510100319. 11398965

[75] Ko YP, Flick MJ. 2016. Fibrinogen is at the interface of host defense and pathogen virulence in *Staphylococcus aureus* infection. Semin Thromb Hemost 42:408–421. doi:10.1055/s-0036-1579635. 27056151 PMC5514417

[76] Beristain-Covarrubias N, Perez-Toledo M, Flores-Langarica A, Zuidscherwoude M, Hitchcock JR, Channell WM, King LDW, Thomas MR, Henderson IR, Rayes J, Watson SP, Cunningham AF. 2019. *Salmonella*-induced thrombi in mice develop asynchronously in the spleen and liver and are not effective bacterial traps. Blood 133:600–604. doi:10.1182/blood-2018-08-867267. 30401709 PMC6474721

[77] Bogomolnaya LM, Santiviago CA, Yang H-J, Baumler AJ, Andrews-Polymenis HL. 2008. “Form variation” of the O12 antigen is critical for persistence of *Salmonella* Typhimurium in the murine intestine. Mol Microbiol 70:1105–1119. doi:10.1111/j.1365-2958.2008.06461.x. 18826410

[78] Konganti K, Ehrlich A, Rusyn I, Threadgill D. 2018. gQTL: a web application for QTL analysis using the collaborative cross mouse genetic reference population. G3 (Bethesda) 8:2559–2562. doi:10.1534/g3.118.200230. 29880557 PMC6071593

[79] Dobin A, Davis CA, Schlesinger F, Drenkow J, Zaleski C, Jha S, Batut P, Chaisson M, Gingeras TR. 2013. STAR: ultrafast universal RNA-seq aligner. Bioinformatics 29:15–21. doi:10.1093/bioinformatics/bts635. 23104886 PMC3530905

[80] Liao Y, Smyth GK, Shi W. 2014. FeatureCounts: an efficient general purpose program for assigning sequence reads to genomic features. Bioinformatics 30:923–930. doi:10.1093/bioinformatics/btt656. 24227677

[81] Love MI, Huber W, Anders S. 2014. Moderated estimation of fold change and dispersion for RNA-seq data with DESeq2. Genome Biol 15:550–521. doi:10.1186/s13059-014-0550-8. 25516281 PMC4302049

[82] Howe KL, Achuthan P, Allen J, Allen J, Alvarez-Jarreta J, Amode MR, Armean IM, Azov AG, Bennett R, Bhai J, Billis K, Boddu S, Charkhchi M, Cummins C, Da Rin Fioretto L, Davidson C, Dodiya K, El Houdaigui B, Fatima R, Gall A, Garcia Giron C, Grego T, Guijarro-Clarke C, et al. 2021. Ensembl 2021. Nucleic Acids Res 49:D884–D891. doi:10.1093/nar/gkaa942. 33137190 PMC7778975

