# Supplementary material for: Elucidating Mechanisms of Tolerance to Salmonella Typhimurium across Long-Term Infections Using the Collaborative Cross
Source: mBio. 2022 Jul 26;13(4):e01120-22. doi: 10.1128/mbio.01120-22 (PMC9426527; doi:10.1128/mbio.01120-22)
Supplement: TABLE S4 [file mbio.01120-22-s0008.pdf]

Supplementary Table 4

Strain	Male	Female	Breeding Facility
CC001/Unc	3	3	TAMU
CC002/Unc	3	3	TAMU
CC003/Unc	3	3	TAMU
CC006/TauUnc	3	3	TAMU
CC011/Unc	6	3	TAMU
CC015/Unc	3	3	TAMU
CC017/Unc	3	4	TAMU
CC024/GeniUnc	3	3	TAMU
CC038/GeniUnc	3	3	TAMU
CC041/TauUnc	4	3	TAMU
CC043/GeniUnc	3	3	TAMU
CC045/GeniUnc	3	2	UNC
CC051/TauUnc	3	3	TAMU
CC053/Unc	3	3	TAMU
CC057/Unc	3	3	TAMU
CC058/Unc	3	3	TAMU
CC072/TauUnc	3	3	TAMU
CC078/TauUnc	3	3	TAMU
