# Supplementary figures and images for: Elucidating Mechanisms of Tolerance to Salmonella Typhimurium across Long-Term Infections Using the Collaborative Cross
Source: mBio. 2022 Jul 26;13(4):e01120-22. doi: 10.1128/mbio.01120-22 (PMC9426527; doi:10.1128/mbio.01120-22)

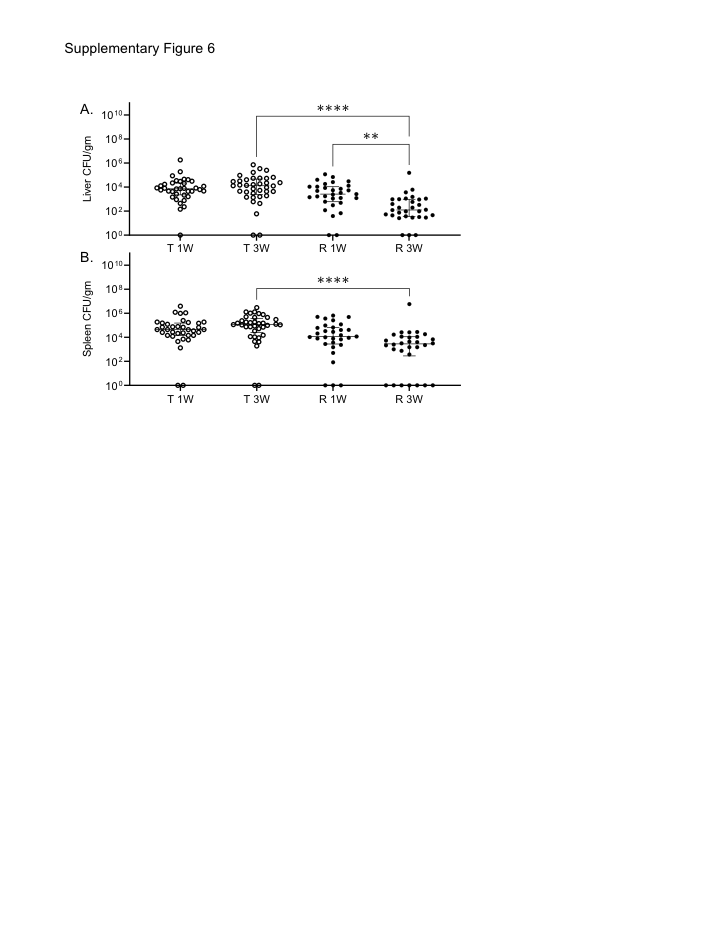

Supplement: FIG S6 [file mbio.01120-22-s0010.tif]

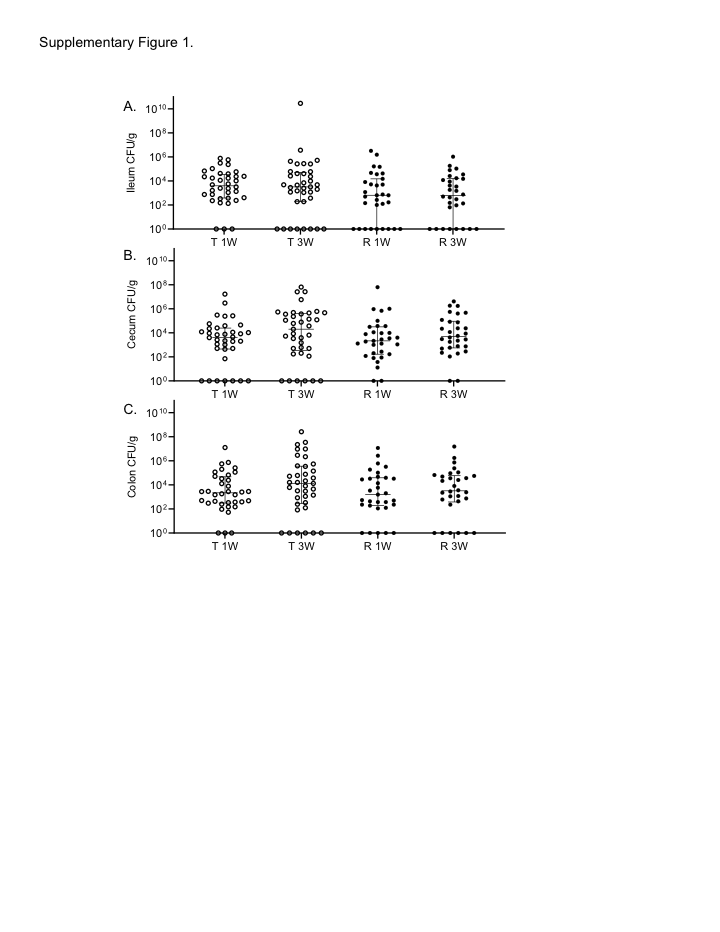

Supplement: FIG S1 [file mbio.01120-22-s0001.tif]

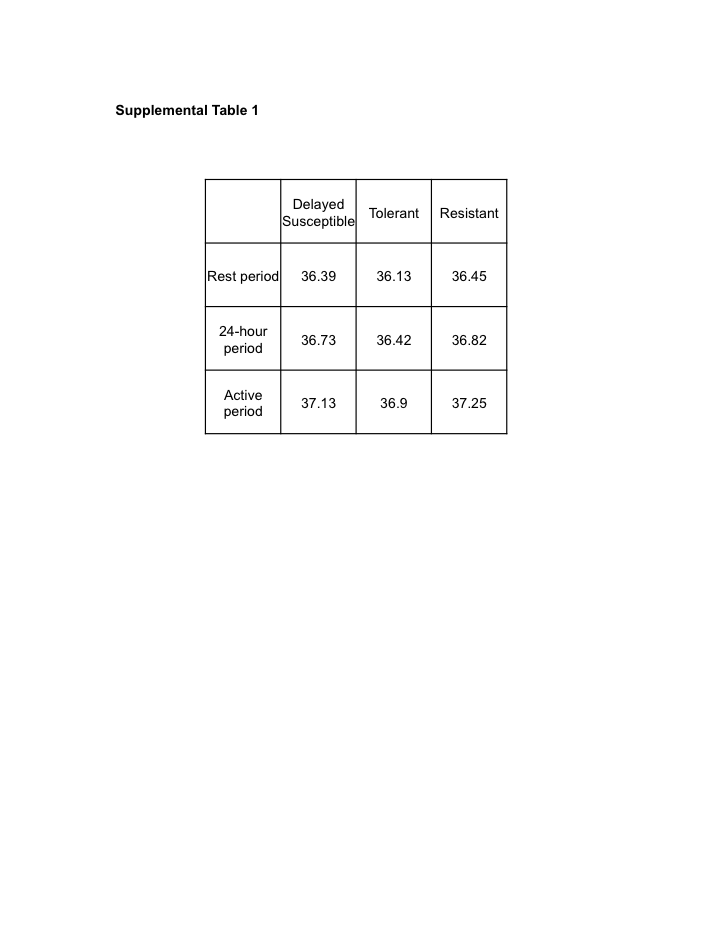

Supplement: TABLE S1 [file mbio.01120-22-s0002.tif]

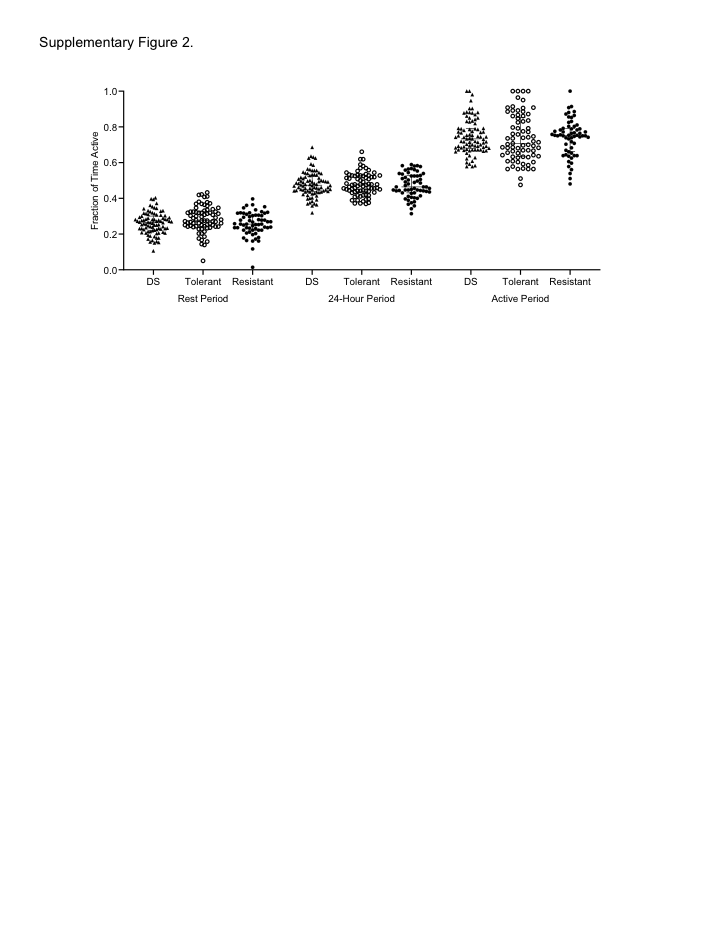

Supplement: FIG S2 [file mbio.01120-22-s0003.tif]

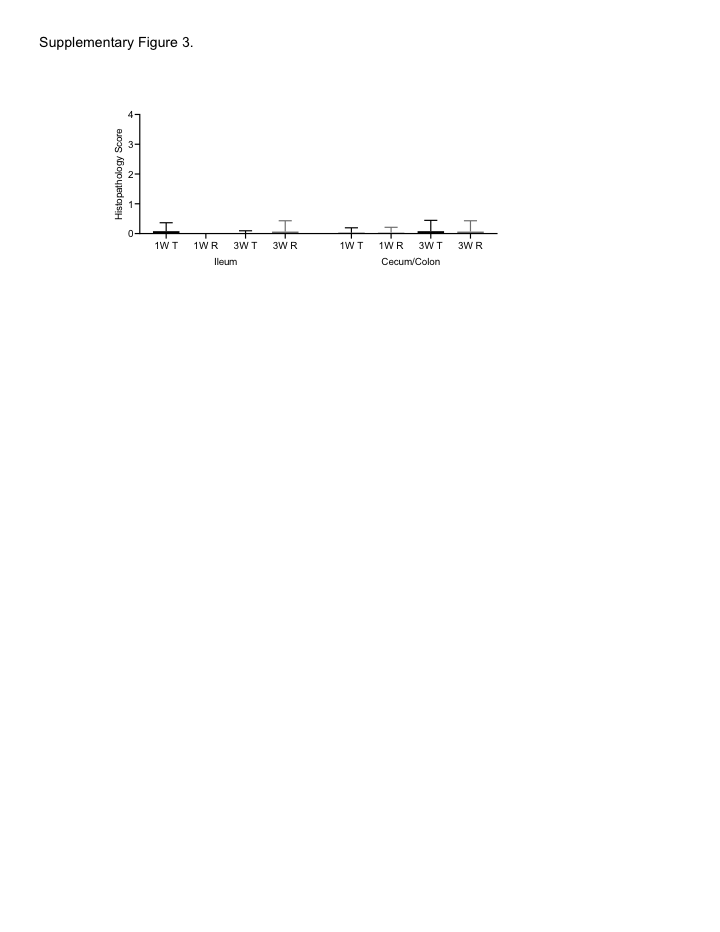

Supplement: FIG S3 [file mbio.01120-22-s0005.tif]

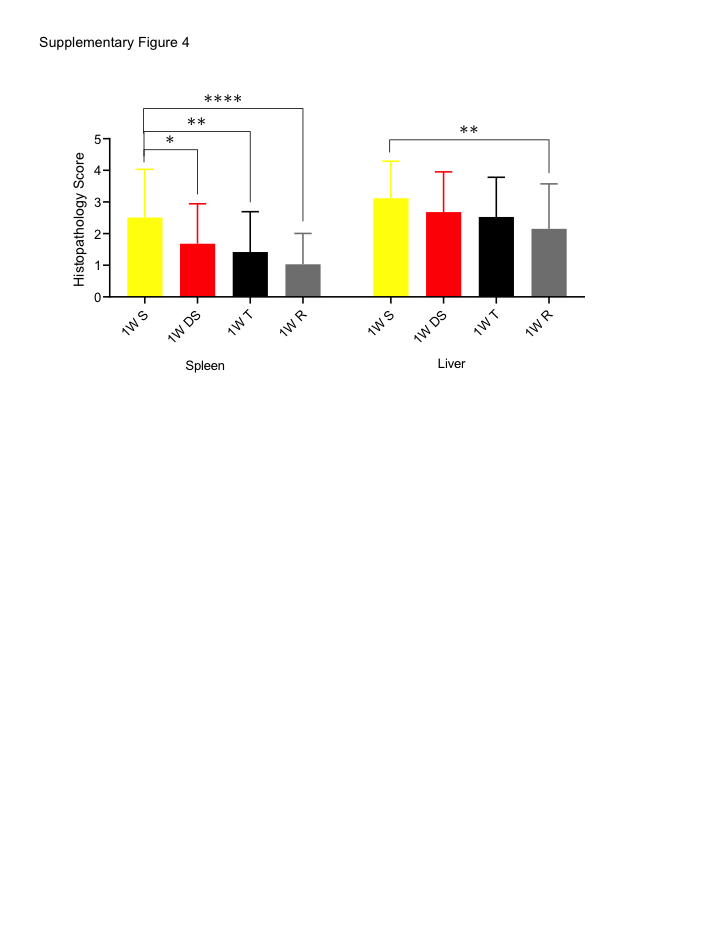

Supplement: FIG S4 [file mbio.01120-22-s0007.tif]

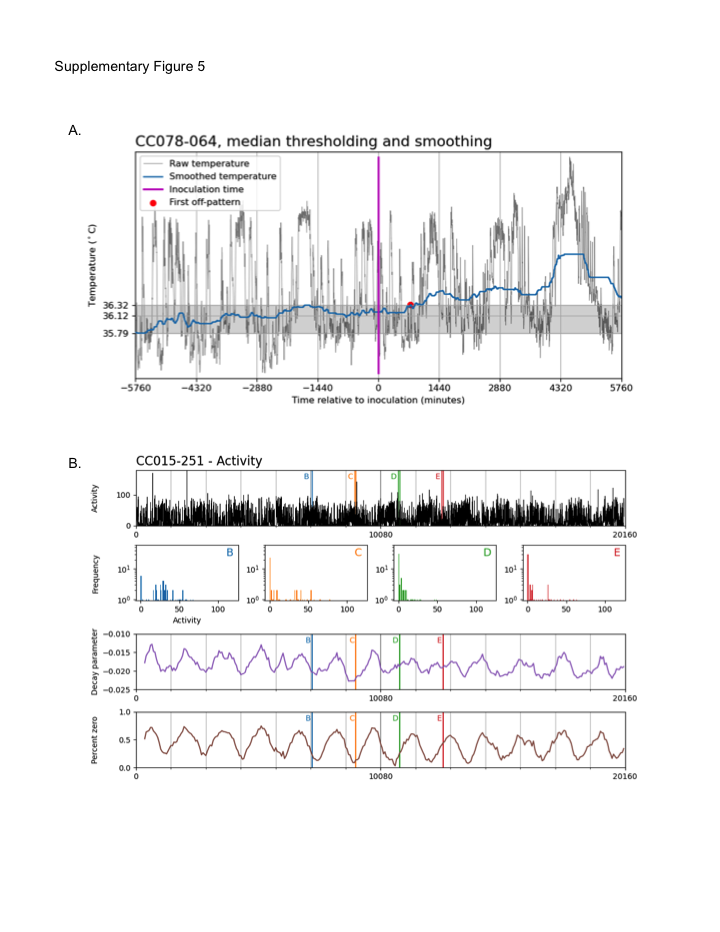

Supplement: FIG S5 [file mbio.01120-22-s0009.tif]
