# Supplementary material for: Elucidating Mechanisms of Tolerance to Salmonella Typhimurium across Long-Term Infections Using the Collaborative Cross
Source: mBio. 2022 Jul 26;13(4):e01120-22. doi: 10.1128/mbio.01120-22 (PMC9426527; doi:10.1128/mbio.01120-22)
Supplement: TABLE S3 [file mbio.01120-22-s0006.pdf]

Supplementary Table 3

A.

Score	Tissue Injury	PMN Infiltrate*	Mononuclear Infiltrate*	Microgranuloma Formation*	Necrosis Cellular or Zonal	Abscessation or Zonal Infarction
0	None	No changes, 0-3	No changes, 0-5	No changes	No changes	No changes
1	Mild	3-5	6-10	1-2	Single cells	None
2	Moderate	6-15	11-80	3-5	Multiple cells	Small, rare
3	Marked	16-150	81-200	6-12	Islands of cells	Moderate, occasional
4	Severe	>150	>200	>12	Broad zones	Massive, common

*Number of cells or microgranulomas per high power field (400X).

B.

Liver Scoring

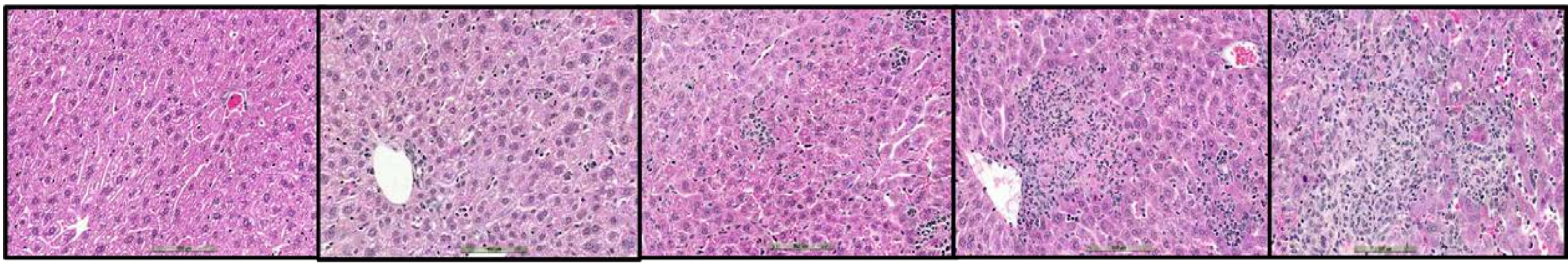

0 1 2 3 4

Spleen Scoring

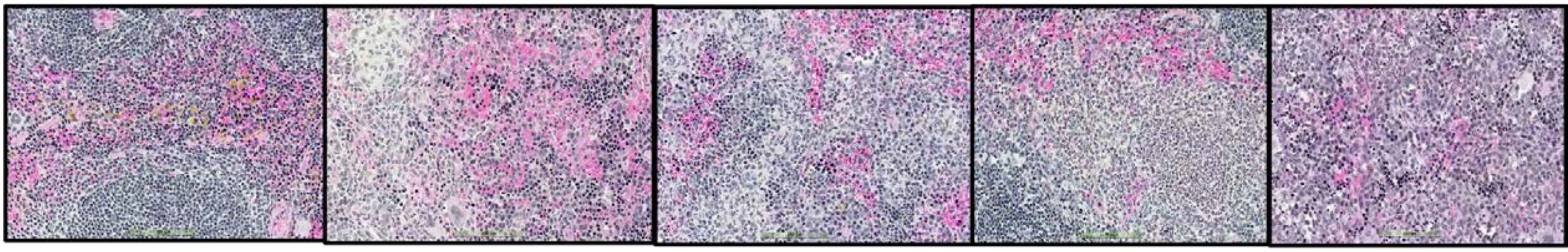

0 1 2 3 4
